# A machine learning approach for predicting CRISPR-Cas9 cleavage efficiencies and patterns underlying its mechanism of action

**DOI:** 10.1371/journal.pcbi.1005807

**Published:** 2017-10-16

**Authors:** Shiran Abadi, Winston X. Yan, David Amar, Itay Mayrose

**Affiliations:** 1 Department of Molecular Biology and Ecology of Plants, Tel Aviv University, Tel Aviv, Israel; 2 Broad Institute of MIT and Harvard, Cambridge, Massachusetts, United States of America; 3 Graduate Program in Biophysics, Harvard Medical School, Boston, Massachusetts, United States of America; 4 Harvard-MIT Division of Health Sciences and Technology, Harvard Medical School, Boston, Massachusetts, United States of America; 5 Blavatnik School of Computer Science, Tel-Aviv University, Tel Aviv, Israel; 6 Division of Cardiovascular Medicine, Department of Medicine, Stanford University, Stanford, CA, United States of America; The University of Texas MD Anderson Cancer Center, UNITED STATES

## Abstract

The adaptation of the CRISPR-Cas9 system as a genome editing technique has generated much excitement in recent years owing to its ability to manipulate targeted genes and genomic regions that are complementary to a programmed single guide RNA (sgRNA). However, the efficacy of a specific sgRNA is not uniquely defined by exact sequence homology to the target site, thus unintended off-targets might additionally be cleaved. Current methods for sgRNA design are mainly concerned with predicting off-targets for a given sgRNA using basic sequence features and employ elementary rules for ranking possible sgRNAs. Here, we introduce CRISTA (CRISPR Target Assessment), a novel algorithm within the machine learning framework that determines the propensity of a genomic site to be cleaved by a given sgRNA. We show that the predictions made with CRISTA are more accurate than other available methodologies. We further demonstrate that the occurrence of bulges is not a rare phenomenon and should be accounted for in the prediction process. Beyond predicting cleavage efficiencies, the learning process provides inferences regarding patterns that underlie the mechanism of action of the CRISPR-Cas9 system. We discover that attributes that describe the spatial structure and rigidity of the entire genomic site as well as those surrounding the PAM region are a major component of the prediction capabilities.

## Author summary

The CRISPR-Cas9 system, a microbial adaptive immune system, was recently exploited for modulating DNA sequences within the endogenous genome in many organisms. This system has emerged as a technology of choice for genome editing with promising therapeutic and research advancements. However, these exciting developments were not paralleled by deep understanding of CRISPR-Cas9 cleavage efficiency. Indeed, while numerous studies have been conducted in order to define better guidelines to determine CRISPR-Cas9 specificity, much ambiguity remains surrounding its mechanism of action. Here, we present a machine-learning based algorithm that was trained on genome-wide experimental data. The algorithm considers a broad range of features that describe different attributes that potentially impact the cleavage efficacy of CRISPR-Cas9 including genomic attributes, RNA thermodynamics, and those concerning sequence similarity. We further found that incorporating the possibility for DNA or RNA bulges play an important role in prediction accuracy. Together, these result in a predictive model that can be used both to predict the cleavage propensity of a new genomic site according to the genomic context, as well as to learn on the importance of different features on CRISPR-Cas9 efficiency and selectivity.

## Introduction

The Clustered, Regularly InterSpaced, Palindromic Repeats (CRISPR), and its associated protein 9 (Cas9) constitute a microbial adaptive immune system that was exploited in recent years for modulating DNA sequences within the endogenous genome in cultured cells and whole organisms [1–6]. The Cas9 endonuclease is directed by a programmable single guide RNA (sgRNA) to induce double strand breaks at specific genomic sites [7,8]. Recognition and cleavage occur via complementarity of a 20-nt sequence within the sgRNA to a genomic site, upstream to a Protospacer Adjacent Motif (PAM) at its 3’-end. Early studies demonstrated that multiple mismatches as well as DNA or RNA bulges can be tolerated [9–15], resulting in cleavage of unintended genomic sites, termed off-targets. This gave rise to devising key considerations for the design of an optimal sgRNA, namely, an efficient guide with minimal off-target effect. Such rules asserted that the number of mismatches should not exceed a specified bound, that mismatches at PAM-proximal positions are more influential than those occurring at PAM-distal positions, that spatially-dispersed mismatches are better tolerated, and that cleavage would not occur at sites that follow PAM sequences other than the canonical NGG (and occasionally NAG) [9–11,13]. However, early studies were not performed on a genome-wide scale as they analyzed off-targets that were pre-selected according to sequence similarity. Thus, such analyses were not designed to detect features outside the scope of pairwise sequence similarity. Subsequently, several experimental methods for unbiased genome-wide profiling of off-targets were introduced, including those based on integration of oligonucleotides into double strand breaks detected by sequencing (GUIDE-Seq) [16–18], high-throughput genome-wide translocation sequencing (HTGTS) [19], direct in situ breaks labelling (BLESS) [20,21], integration-deficient lentiviral vectors (IDLV) [22], and in-vitro nuclease-digested whole-genome sequencing (Digenome-seq) [23,24]. These studies demonstrated that CRISPR off-targets can be located at unexpected sites, such as sites that harbor alternative PAM sequences, sites that contain a large number of mismatches, and off-targets that were cleaved at higher frequencies than the intended on-targets. Thus, it is becoming clear that an intricate set of attributes play a role in CRISPR-Cas9 function.

To date, several computational methods for sgRNA design were developed based on different design rules [25–42]. For example, CCTop [25] considers the distance of the mismatch from the PAM site when evaluating the specificity of candidate sgRNAs, ‘Optimized CRISPR Design’ [26] incorporates a position-specific mismatch penalty and additionally considers the spatial distribution of mismatches, and the CFD score [28] penalizes each mismatch according to its specific substitution type and position. Importantly, while these and other widely-used methods have been developed based on empirical data, they mostly neglect the genomic context surrounding the target sequence and instead focus on predicting off-target effects for a given sgRNA using basic sequence features [25,29,34,35,43].

Here, we introduce CRISTA, a novel methodology based on the machine learning paradigm for predicting the cleavage propensity of a genomic site by a given sgRNA. The method accounts for the possibility of bulges and incorporates a wide range of features encompassing those that are specific to the genomic content, features that define the thermodynamics of the sgRNA, and features concerning the pairwise similarity between the sgRNA and the genomic target. We show that CRISTA achieves a higher predictive accuracy than widely-used alternatives. We further examine our approach using a leave-study-out cross-validation procedure, thereby demonstrating that the predictive model represents general patterns of the cleavage machinery across different detection techniques. In addition to its predictive value, our method suggests additional information on the underlying mechanism of action of the CRISPR-Cas9 system, including attributes that were previously overlooked.

## Methods

### Data assembly

The training dataset was assembled from published data obtained using several genome-wide unbiased methods for CRISPR-Cas9 cleavage sites profiling: GUIDE-Seq, HTGTS, and BLESS [16,17,19–21]. These datasets are termed hereafter Tsai [16], Kleinstiver [17], Frock [19], Ran [20], and Slaymaker [21]. The data in these studies are composed of collections of experimentally verified genomic targets throughout the genome, such that each target is denoted with the frequency of cleavage by a given sgRNA. We note that additional systems for cleavage sites detection are available, but these are not compatible with our objective to reveal genomic effects on CRISPR efficacy. For example, Digenome-Seq [23,24] does not provide cleavage frequencies *in-vivo*; the integrase-defective lentiviral vectors (IDLV) method can be used to detect off-targets *in-vivo*, but does not provide their cleavage frequencies [22]. Furthermore, a number of studies employed targeted sequencing approaches [15,22] to examine the cleavage frequencies of several genomic sites that were pre-selected based on prior deductions, and thus are lacking the information at the genomic scale. In total, data from five genome-wide studies were assembled, spanning 33 collections of sgRNAs and their respective targets obtained from 25 unique sgRNAs (S1 and S2 Tables). Combined, these sgRNAs cleaved 872 and 491 genomic targets across the genome before and after data filtration, respectively (see “Training dataset assembly” below). We refer to these data as the set of cleaved sites. Notably, the collection of targets was obtained from multiple methodologies and under different experimental conditions, hence, their reported cleavage efficiencies are not comparable and were thus transformed to a common scale. To this end, for each platform we extracted the set of sgRNAs that are in common with those from Tsai et al. [16], which is the most inclusive dataset. We then fitted the cleavage frequencies of the mutual targets of each study and Tsai data using linear regression. The inferred regression parameters were then used to transform the rest of the data obtained from the respective study (for more details see S1 Text, S1 and S2 Figs).

### Pairwise alignment to account for bulges

In an initial exploratory phase, we observed that the pairings of the sgRNAs and the corresponding genomic sites, as originally reported, occasionally contained an exceedingly large number of mismatches. For example, 243 out of 872 sites retained five to ten mismatches, 22 of these had cleavage frequencies that were ranked among the highest 25% (S2 Table). This is in contrast to previous reports that showed that observing more than five mismatches is highly unlikely [9–11,13]. While these studies mainly concentrated on the number of mismatches, more recent evidence suggested that DNA/RNA bulges are also possible [12], and these can be represented as indel events in the context of sequence alignment. To account for this possibility and for additional specific characteristics of the CRISPR-Cas9 system, we modified the Needleman-Wunch pairwise alignment algorithm [44] to include two additional components: (i) Up to three single gaps are allowed over the whole alignment–a bound that was rarely met (and was never exceeded) in the set of cleaved sites but was necessary in order to detect potential off-targets in a computationally efficient manner. (ii) Since three gaps are allowed, each 20nt long genomic target is extended or shortened by up to three nucleotides, and the best pairwise alignment score over seven independent alignments between the DNA site (of length 17-23nt) to the corresponding sgRNA is selected.

The pairwise alignment is determined by the match, mismatch, and gap parameters, such that a bulge (i.e., a gap), would be preferred over a mismatch only if the penalty paid for its insertion is compensated by the matches it induces. To determine the ideal parameters for pairwise alignment, we repeated the alignment procedure by ranging over different combinations of parameter values. The parameters that resulted in the maximal averaged squared Pearson correlation coefficient (*r*^2^) between the cleavage intensities and the pairwise-alignment scores were then selected. In this optimization procedure, targets of exact match were removed since these always result in the highest possible score and could shift the obtained *r*^2^ values. This procedure was performed either across the whole dataset, as well as for the partial data used in cross-validation (see below).

A total of 119 targets, as obtained from the original studies, follow a non-NGG PAM (54 in Tsai data, 31 in Kleinstiver data, 34 in Frock data). Originally, the coordinates of the cleaved sites were detected by matching sequences to the reference genome while considering mismatches only. Thus, for example, if bulges are disregarded, a possible DNA-bulge upstream to a canonical PAM would be interpreted as a target with a non-canonical PAM. The introduction of gaps in the alignment allowed us to correct such instances. Hence, we re-evaluated the position of all non-NGG targets by shifting the PAM genomic coordinates 2-nt downstream or upstream in search for an NGG PAM or, if one did not exist, an NAG PAM at closest proximity. If none were found, the original PAM was preserved.

### A machine learning algorithm for predicting cleavage propensity

We developed CRISTA, a tool for predicting the cleavage propensity of potential genomic targets given a specified sgRNA. CRISTA is based on learning a regression model using the Random Forest algorithm, and further allows the examination of the importance of features that determine the variation of cleavage efficiency. The development of a machine learning algorithm relies on (i) the assembly of a training dataset that encompasses a range of data inputs, and (ii) the incorporation of a set of features that can be used to predict cleavage efficiencies. The utility of the learning framework to distinguish between cleaved and uncleaved sites was also examined within a classification learning scheme (as opposed to a regression model). As the results were generally similar, those obtained with the regression model are presented throughout (see Discussion).

#### Training dataset assembly

To enhance the learning process, a set of uncleaved sites was assembled, representing sites that were not cleaved by each sgRNA. Theoretically, excluding the set of cleaved sites of each sgRNA, the whole genome can be taken to represent the uncleaved set. Because this set is too broad for meaningful analysis, we included only uncleaved genomic sites with sufficient sequence complementarity to each sgRNA. To this end, genomic sites were extracted from the UCSC genome assembly [45] as follows: each sgRNA from the dataset was aligned to sites that follow NGG or NAG motifs in the genome, according to pairwise sequence alignment as described above. Then, sites with an alignment score greater than 14.75 (as 95% of the cleaved instances, which on average have 16.7 matched bases) were retained for further analysis. The number of sites in the uncleaved sets varied from 3,000 to 70,000 per sgRNA. We note that this procedure might introduce some noise for targets in which the reference genome is not identical to the genome of the cell-line used in the experimental systems.

The combined training dataset was assembled from the experimentally validated cleavage sites together with the uncleaved sites. The dependent variable was the cleavage efficiencies reported in each study (following the linear transformation; S1 Text) for the cleaved samples and zero for the uncleaved samples. Duplicated collections of samples (namely, targets of sgRNAs that were tested in multiple experiments) were filtered out, while retaining a unique set, corresponding to the validated targets in Tsai data, as it is the most comprehensive study. Additionally, since Frock data was found to be incompatible with the rest of the training set (see Results), this dataset was removed. Notably, the number of samples in the set of uncleaved sites was much higher than the set of cleaved sites and thus combining these two sets into a single training datasets would have resulted in a sharp bias towards the set of uncleaved sites. To allow the incorporation of a large repertoire of uncleaved samples without biasing towards them, we under-sampled the majority class and over-sampled the minority class as suggested by Chawla et al. [46]. Each set of cleaved samples (targets that correspond to a single sgRNA) was oversampled using bootstrapping, thus introducing a subset twice the size of the original one, and an equal-sized set of uncleaved samples was randomly chosen. We repeated this process and averaged the results over executions of the algorithm on 100 sampled datasets.

#### Predictive features

We computed a wide range of possible explanatory attributes that range from features that are specific to the target site (e.g., the type of the PAM sequence, nucleotide composition and GC content, chromatin structure, CpG islands, gene expression levels of coding regions), to those that are specific to the sgRNA (e.g., sgRNA secondary structure), to those concerning the similarity between the sgRNA and the target (e.g., number and spatial distribution of mismatches and bulges). For a full description of the features and their extraction procedures, see S3 Table.

#### Implementation and availability

Given the training dataset and a set of features, we implemented CRISTA using the RandomForestRegressor, implemented in the python scikit-learn module [47,48]. The score provided by CRISTA, essentially represents the log number of sequencing reads identified by GUIDE-seq (divided by the maximal number), which in turn represents a proxy for the cleavage frequency, as was shown by Tsai et al. [16]. This score is hereafter referred to as the inferred cleavage propensity. Notably, this score is continuous, and thus does not provide a binary classification for potentially cleaved and uncleaved sites by a given sgRNA. Yet, such categorizations could be practically needed by users. The scores predicted by CRISTA for the observed cleaved sites can be used to set a strict or a lenient threshold. This can be determined according to either the training dataset or the data used for external validation [15,22]. For example, 95% of the cleaved sites in the cleaved dataset used by CRISTA obtained a score higher than 0.12, while 50% surpassed the score of 0.4. In the validation dataset, these thresholds were 0.39 and 0.54 for 95% and 50%, respectively.

The CRISTA algorithm is available for online use at http://crista.tau.ac.il/ and the source code is available for offline use. The server provides three optional entry points: (1) given a set of nucleotide targets with the corresponding sgRNAs, CRISTA provides the predicted cleavage score for each pair. The genomic targets can be given by their extended genomic sequence or genomic coordinates. (2) Given a specified nucleotide sequence, CRISTA identifies all potential targets within it (i.e., those followed by ‘NGG’) and ranks these according to the predicted cleavage score. (3) Given an sgRNA and a specified genome (currently 230 genome assemblies are supported encompassing vertebrates, plants, yeast, insects, and deuterostomes [49,50]), CRISTA detects possible off-targets throughout the genome. As opposed to most currently available alternatives, the off-targets detected by CRISTA also include DNA/RNA bulges. A comprehensive detection of off-targets using the pairwise alignment approach described above is computationally demanding. Thus, the search in the online web server is based on an approximate search using BWA-ALN [51] with the following parameters: “-N -l 20 -i 0 -n 5 -o 3 -d 3 -k 4 -M 0 -O 1 -E 0”. This identifies all targets with up to four mismatches and/or gaps in the 20-nt matching region. Increasing this number to five, resulted in exceedingly long running times.

### Assessing algorithm performance

**Fig 1:**
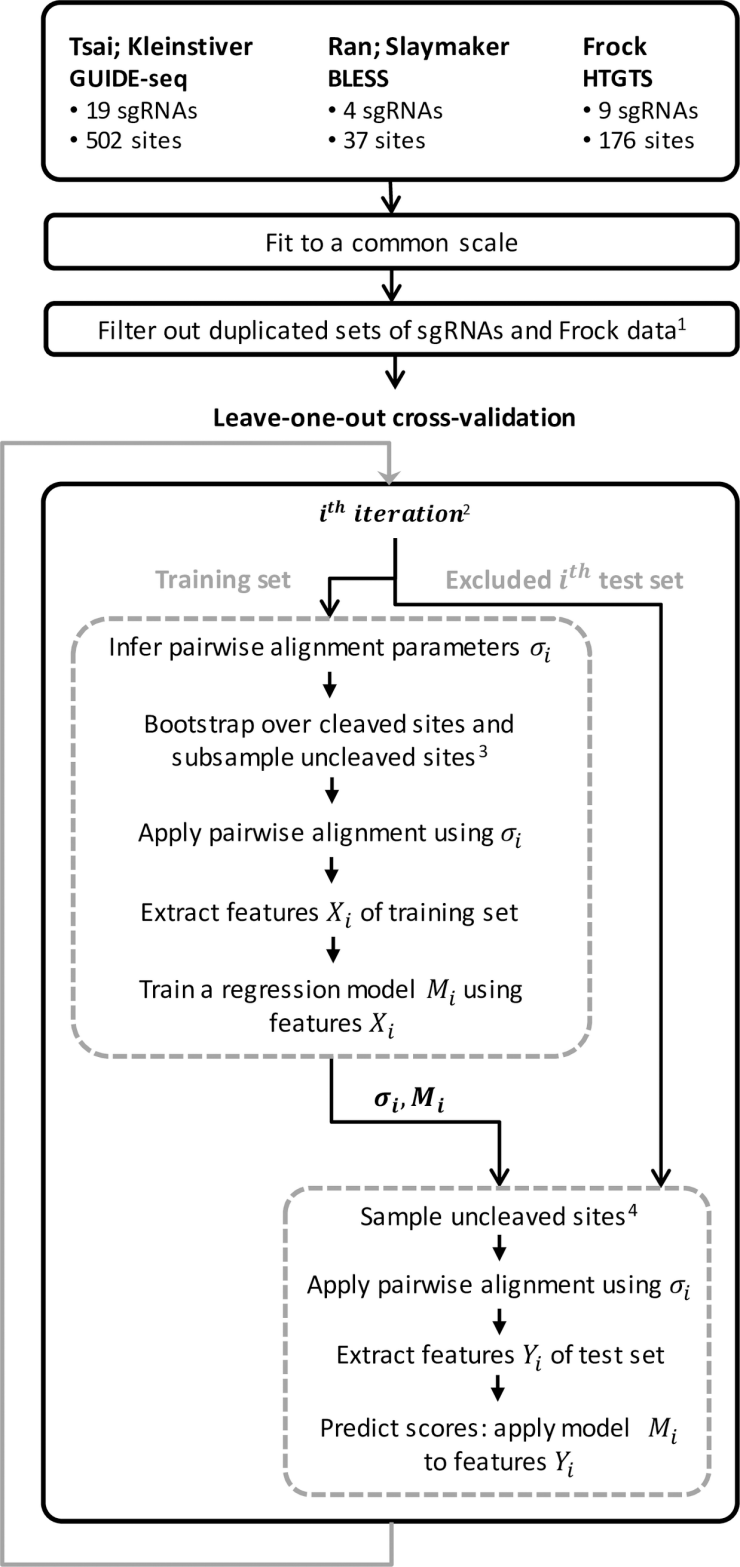
Schematic flow of the cross-validation procedures. The main components of the learning pipeline for the leave-one-sgRNA-out and leave-study-out cross-validation procedures are presented. ^1^ This step was applied to the leave-one-sgRNA-out procedure only. ^2^ In each iteration, the samples of a single sgRNA (in the case of the leave-one-sgRNA-out procedure) or all samples from a single study (in the case of leave-study-out) were excluded from the training data and used as a test set. The algorithm was trained on the rest of the data. ^3^ Each set of cleaved samples (targets that correspond to a single sgRNA) was oversampled using bootstrapping, thus introducing a subset twice the size of the original one, and an equal-sized set of uncleaved samples was randomly chosen. ^4^ For each original set of cleaved samples in the test set (targets that correspond to a single sgRNA), an equal-sized set of uncleaved samples was randomly chosen.

We evaluated the prediction performance of CRISTA using two cross-validation procedures (Fig 1). We devised a leave-one-sgRNA-out procedure, such that in each iteration the samples of a single sgRNA were excluded and used as a test set. The algorithm, trained on the rest of the data, was then used to predict the cleavage probabilities for the test set. Each iteration of the cross-validation consisted of a preliminary step: the pairwise alignment parameters were first optimized as previously described using the training set only, and then were used to re-compute the pairwise alignment features for the training and the test sets. Similarly, we used a leave-study-out cross-validation strategy such that in each iteration all samples from a single study were excluded from the training data and used as a test set (note that Tsai data were divided to two datasets, S1 Text). Whereas the training dataset of CRISTA—which was used in the leave-one-sgRNA-out procedure and for all reported comparisons—did not include redundant sgRNAs to avoid overfitting of the model to the data, here we calculated the performance scores separately for sgRNAs that were uniquely inspected in one study (termed ‘unique guides’), and sgRNAs that were analyzed in more than one study (termed ‘common guides’; S1 Table).

Several metrics (squared Pearson correlation coefficient and the area under the Receiver Operator Characteristics and Precision-Recall curves), were used to evaluate the performance of CRISTA and to compare it to three widely used alternatives; CCTop [25], the function for scoring single off-targets used in the online tool ‘Optimized CRISPR Design’ [26] (hereafter termed *OptCD*), and the CFD score [52]. The performance evaluation reported throughout was computed over the original set of cleaved sites for each sgRNA (without bootstrapping as was performed in the training set), and an equally-sized sample of uncleaved sites (see Results for the effect of this sample size on the performance evaluation).

### Identifying a succinct set of influential features

The Random Forest algorithm computes the relative contribution of the examined features to the regression model, termed *feature importance*. When the entire set of features is examined (S3 Table), some features may receive seemingly low importance values due to the presence of a correlated feature (e.g., the pairwise alignment score and the number of mismatches). To learn on the independent importance of the various features, we reduced the number of features by applying a forward selection procedure. Features were added iteratively by examining the performance of the leave-one-sgRNA-out cross-validation procedure for incremental sets of features. First, we tested which feature provides the highest Pearson *r*^*2*^ when examined independently. Then, in each iteration, the feature that increased the *r*^*2*^ the most was adjoined to the set. This procedure was repeated for 15 iterations. Random Forest was then applied to the resulting set of features and the relative importance of each feature was extracted.

## Results

### Accounting for bulges

The introduction of gaps to the pairwise sequence alignment affected 18% of the targets in the training dataset, such that 87 of 491 sites contain 1.1 bulges on average (or an average of 1.23 in 175 out of 872 sites if considering the full dataset; S2 Table). This resulted in *r*^2^ = 0.34 (squared Pearson correlation coefficient between the pairwise alignment score and the observed cleavage frequencies) averaged over the sgRNAs datasets compared to *r*^2^ = 0.27 when gaps are not allowed. The optimized parameter values were 1 for a match, 0 for a mismatch and -1.25 for a gap (S3 Fig). We note that although mismatches are not explicitly penalized, matches are still awarded and so longer complementarity is generally preferred. Following this procedure, the number of mismatches was reduced from an average of 3.64 to 3.36 per target, such that six mismatches became very rare (S4 Fig). Reconsidering the PAM locations, such that NGG or NAG PAMs were found, resulted in a shift of 33, 17, and 22 instances (out of 54, 31, and 34 targets with rare PAMs) of Tsai, Kleinsteiver, and Frock data, respectively (S2 and S4 Tables). Notably, the pairwise similarity score explains merely 34% of the observed variation among the cleaved sites, which motivated us to integrate additional features in the prediction process.

### A machine learning algorithm for predicting cleavage propensity

**Fig 2:**
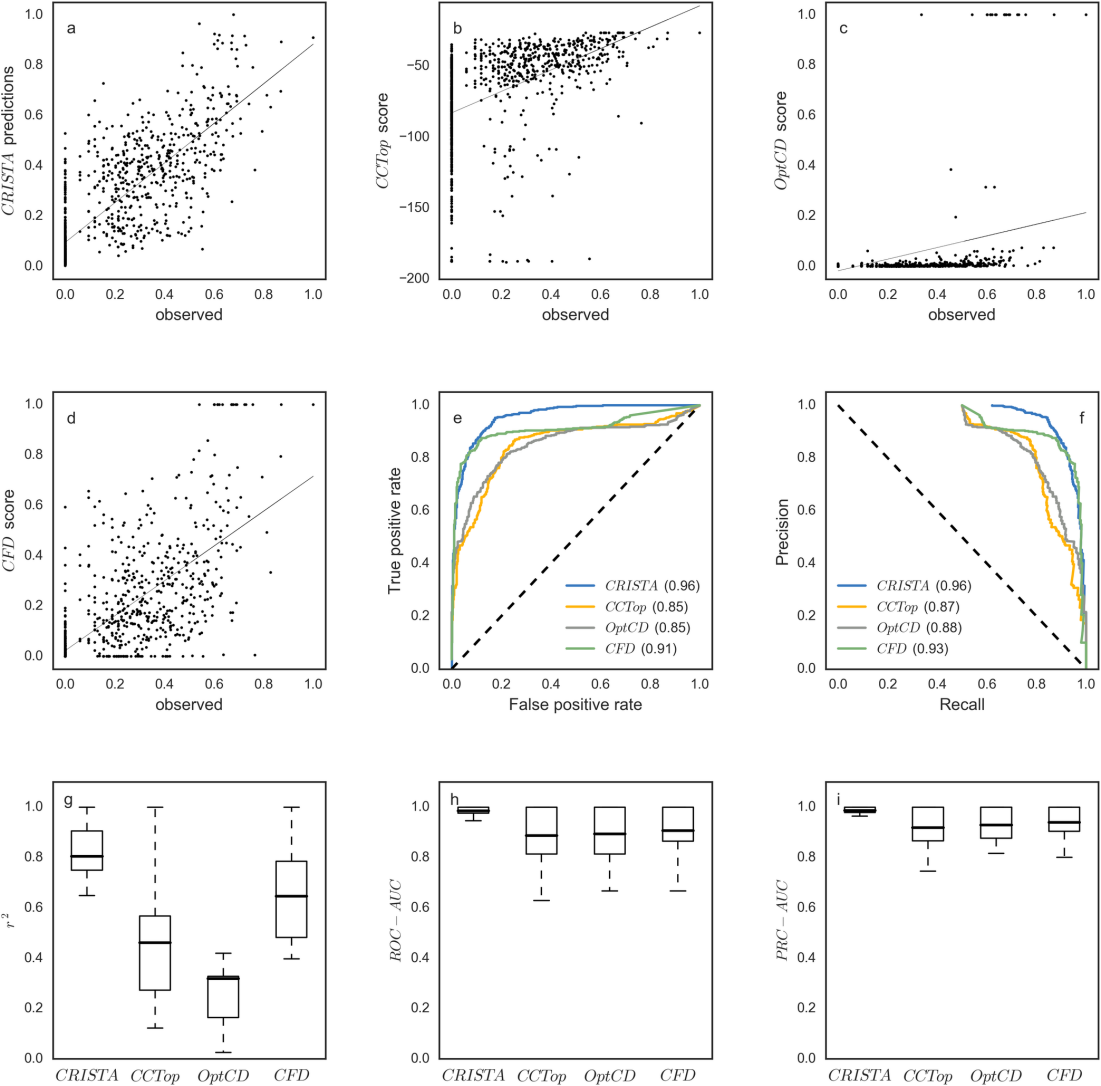
Comparison of four prediction algorithms on the assembled dataset. (a-d) Pearson correlation coefficient computed over all the samples in the dataset. The horizontal axis represents the scaled observed values published in the experimental studies, and the vertical axis represents the scores predicted by: (a) CRISTA applied using cross-validation (*r*^2^ = 0.65), (b) CCTop (*r*^2^ = 0.23), (c) OptCD (*r*^2^ = 0.13), (d) CFD score (*r*^2^ = 0.52). (e) Receiver Operator Characteristics curves computed over all the samples in the test dataset: CRISTA (AUC = 0.96), CCTop (AUC = 0.85), OptCD (AUC = 0.85), CFD score (AUC = 0.91). Positives and negatives represent cleaved and uncleaved sites, respectively. True (and false) positives rate is computed as the true-positives (false-positive) number divided by the number of positives (negatives). (f) Precision-Recall curves computed over all the samples in the dataset: CRISTA (AUC = 0.96), CCTop (AUC = 0.87), OptCD (AUC = 0.88), CFD score (AUC = 0.93). Precision is computed as the true-positive number divided by the sum of true-positives and false-positives. Recall is computed as the true-positives number divided by the positives number. (g) Pearson correlation coefficient computed for each sgRNA: CRISTA (averaged *r*^2^ = 0.80, *sd* = 0.13), CCTop (averaged *r*^2^ = 0.46, *sd* = 0.22), OptCD (averaged *r*^2^ = 0.32, *sd* = 0.28), CFD score (averaged *r*^2^ = 0.65, *sd* = 0.28). (h) Receiver Operator Characteristics curves computed for each sgRNA: CRISTA (averaged AUC = 0.99, *sd* = 0.02), CCTop (averaged AUC = 0.86, *sd* = 0.13), OptCD (averaged AUC = 0.9, *sd* = 0.12), CFD score (averaged AUC = 0.9, *sd* = 0.11). (i) Precision-Recall curves computed for each sgRNA: CRISTA (averaged AUC = 0.99, *sd* = 0.02), CCTop (averaged AUC = 0.92, *sd* = 0.09), OptCD (averaged AUC = 0.93, *sd* = 0.07), CFD score (averaged AUC = 0.94, *sd* = 0.06). Mean values are marked with horizontal lines. The whiskers reach 1.5 times past the first and third quartiles.

We devised CRISTA, a machine learning methodology that is based on the Random Forest regression model [47,48]. CRISTA was trained on several genome-wide experimental studies and combines a large set of explanatory features, to compute the cleavage propensity of a DNA target by an sgRNA. The resulting regression function of CRISTA is composed of a complex interaction between the incorporated features as represented by a set of decision trees. We evaluated the prediction performance of CRISTA in a leave-one-sgRNA-out cross-validation procedure, and compared it to the alternative tools. First, we calculated the squared Pearson correlation coefficient (*r*^*2*^) between the experimentally observed cleavage frequencies and the predictions. The scores that were predicted in the cross-validation conformed to the observed values with an *r*^*2*^ of 0.65. In comparison, *OptCD* produced an *r*^*2*^ of 0.13, the scores obtained using CCTop resulted in an *r*^*2*^ of 0.23, while the CFD score correlated best out of the three commonly-used alternatives with an *r*^*2*^ of 0.52 (Fig 2A–2D, S5 Table). A similar trend regarding the relative performance of the four scoring functions was obtained when Spearman rank correlation was computed (Spearman rho coefficients for CRISTA, OptCD, CCTop, and the CFD score were 0.81, 0.66, 0.64, and 0.74 respectively).

Second, the receiver operating characteristic (ROC) curve was used in order to compare the abilities of the tools to discriminate between experimentally cleaved and uncleaved sites (thus, for this performance evaluation we treat these as the positive and negative sets, respectively), as measured by the area under the curve (AUC, values closer to 1.0 represent better predictions). To this end, we used the predicted scores as thresholds to delineate positives and negatives for the ROC calculation. Using this measure a similar trend was observed regarding the relative accuracy of the prediction methods (Fig 2E). CRISTA had the highest AUC score of 0.96 followed by the CFD score (AUC = 0.91), OptCD (AUC = 0.85) and CCTop (AUC = 0.85). Noticeably, all methods received high AUC scores, but this could be due to the large number of uncleaved sites that were included in the dataset. Hence, we further compared the ability to detect and to rank among the positive samples, as measured using the area under the Precision-Recall curve (PRC-AUC). Similar to the ROC curve, PRC-AUC values closer to 1.0 indicate highly successful predictions. Again, the ability of CRISTA to rank among the cleaved samples was favorable to the other three methods, with a PRC-AUC of 0.96, compared to 0.93, 0.88, and 0.87 that were obtained using the CFD score, OptCD, and CCTop, respectively (Fig 2F).

The accuracy measures described above were computed while combining the predicted values across the whole dataset. Additionally, we tested whether the alternative prediction tools are consistent, that is, whether or not similar accuracies are obtained across different sgRNAs. The accuracy of CRISTA was found to be the most persistent across distinct sgRNA datasets, with an averaged *r*^*2*^ of 0.8 and a standard deviation of *sd* = 0.13. In comparison, the CFD score, OptCD, and CCTop obtained averaged *r*^*2*^ values of 0.65 (*sd* = 0.2), 0.32 (*sd* = 0.28) and 0.46 (*sd* = 0.25), respectively (Fig 2G; similar results were obtained when considering the ROC-AUC and PRC-AUC measures, Fig 2H–2I; averaged Spearman correlation coefficients were 0.88, 0.77, 0.76, and 0.72, respectively). Notably, while the uncleaved sites are an integral part of the learning process, as well as for assessing the accuracy of the different tools, the reported metrics could be biased to those sites with a “0” cleavage frequency. To examine to what extent the set of uncleaved sites affects the results, the averaged *r*^*2*^ was re-computed while altering the sample size of this set from 100% to 0% (relative to the size of the set of cleaved sites). Our results show that reducing the sample size has little impact on the relative success of the different tools. While the obtained *r*^*2*^ values decrease with lower proportion of uncleaved sites, the ones achieved by CRISTA are still better than the other alternatives (evidently, the decline for CRISTA is shallower than that obtained by the CFD score, which is the second-ranked tool; S6 Table).

### Accuracy across different detection techniques

**Fig 3:**
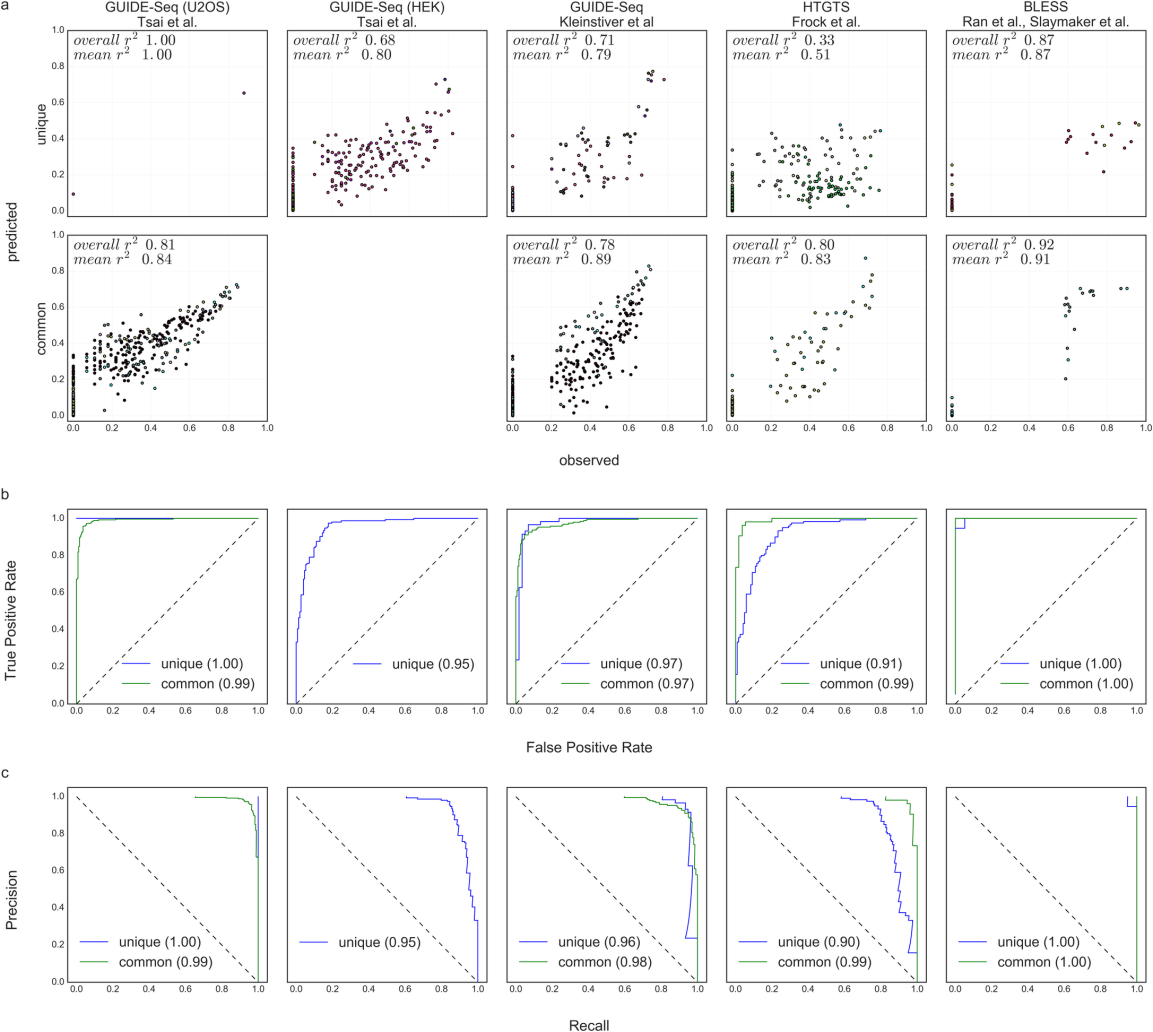
Accuracy across different studies in a leave-study-out cross-validation. (a) Observed cleavage intensities versus predicted intensities. The top and bottom rows represent the nuclear targets of the ‘unique guides’ and ‘common guides’, respectively. Pearson ***r***^**2**^ values are shown; "overall" represents the correlation calculated by taking all points, and "mean" is the average correlation calculated for each sgRNA individually. Different colors represent nuclear targets of different sgRNAs. (b, c) ROC and PRC curves. The ‘unique guides’ and ‘common guides’ of each study are represented by different curves. AUC values are denoted in the legend. Each column corresponds to a single experimental platform.

The learning dataset of CRISTA combines data from three experimental methodologies for genome-wide profiling of CRISPR cleavage sites with some of these applied in multiple experimental settings. Thus, we used a leave-study-out cross-validation procedure to examine whether the accuracy of CRISTA is dependent on a single platform that dominates the learning dataset. This allowed us to examine both the compliance of the different methods, and the performance of the predictive model on data that is similar to the training set (the set of *common guides*, see Methods, S1 Table) and on new data (*unique guides*). Our results demonstrated that, with the exception of the data by Frock et al., the different experimental procedures comply with one another (Fig 3, S7 Table). That is, when each study was used as a test set, without being included in the training set, the prediction made by CRISTA resulted in *r*^*2*^ higher than 0.8, and ROC-AUC and PRC-AUC values close to 1. In addition, the prediction accuracies of the common guides did not overwhelmingly exceed those of the unique guides, indicating that the prediction of cleavage efficiencies was accurate not only when the predictor was trained on similar sgRNAs as in the test data, but also when it was applied to unfamiliar data. Our analysis further demonstrated that the datasets obtained with HTGTS for unique sgRNAs are not comparable with those obtained with the other platforms. Therefore, Frock data was eliminated from the training dataset of CRISTA.

### The contribution of the uncleaved sites to the learning procedure

A central component of the learning procedure implemented in CRISTA is the ample amount of data contained within the set of uncleaved sites as it conceals significant information regarding the features that hinder CRISPR-Cas9 action. Yet, such wealth of information was generally ignored by previous studies that aimed at devising rules regarding CRISPR-Cas9 specificity. To examine whether the enhanced accuracy achieved by CRISTA, as compared to other tools, stems from the inclusion of a large set of uncleaved sites, we repeated the leave-one-sgRNA-out procedure while retaining only the set of cleaved sites in the training set. The accuracy achieved by this model, referred to as CRISTA^+^, was substantially lower compared to CRISTA when trained on the whole dataset (S5 Fig), and is more similar to the one obtained using the CFD score.

### Features importance

**Fig 4:**
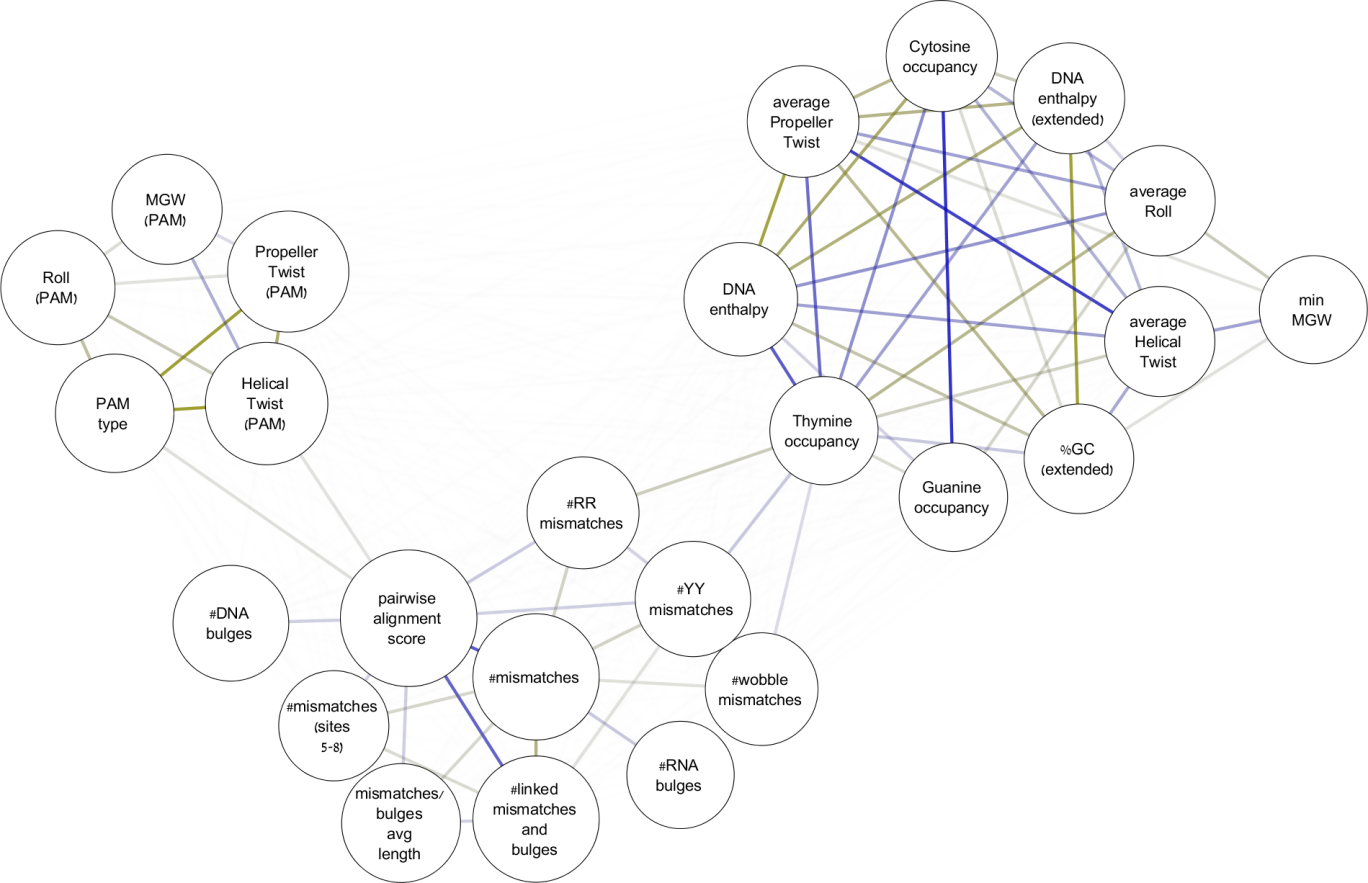
Features importance. Clustering of top-ranked features and their relative importance. The nodes sizes represent the feature importance as calculated by CRISTA. Edges transparencies represent correlation such that strongly correlated features are connected by darker edges. Yellow and blue edges represent positively and negatively correlated features respectively. Abbreviations: YY- mismatches of type pyrimidine-pyrimidine; RR–mismatches of type purine-purine; MGW–minor groove width; ‘#’ represents counts (for further explanations of the features, see S3 Table). The graph was produced with Cytoscape [55] using the pairwise correlation for every pair of features and their importance scores.

Beyond prediction capabilities, the learning process provided the opportunity to systematically learn the attributes that are most important for Cas9 function. When examining the entire set of features (S3 Table), three clusters emerged among the top first 25 (Fig 4): (i) features concerning the pairwise similarity between the sgRNA and the DNA site. Besides the pairwise alignment score, this cluster included the number of mismatches, the number of RNA/DNA bulges, and the mismatches types (i.e., whether they are transition, transversion, or wobble); (ii) features concerning the nucleotides content of the 20-nt site and its adjacent genomic region. These included the GC content, DNA enthalpy (a proxy for the DNA duplex stability [53]), and several measures that describe the spatial structure of the DNA including the minor groove width and the bending stiffness [54]; (iii) features concerning the PAM site and the surrounding nucleotides. These included the PAM type (i.e., NGG or NAG) and DNA geometry scores calculated in and around this region (i.e., NNGGNN if considering the canonical PAM).

**Fig 5:**
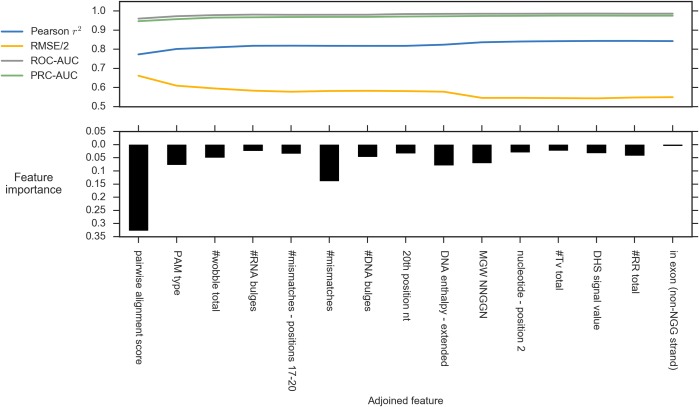
Forward selection results. The top plot represents the ROC-AUC, PRC-AUC, *r*^*2*^, and root mean square error (RMSE) following the addition of every feature from left to right. The bars represent feature importance, i.e., the contribution of every feature to the prediction accuracy as computed by the Random Forest algorithm. The RMSE is divided by two for visualization.

To learn about the features that are most important for prediction, and to reduce the redundancy introduced by correlated features, we obtained a succinct group of 15 elementary features using a forward selection process for which the relative importance was extracted (Fig 5, for the accuracy measurements achieved for the first 30 selected features see S8 Table). As expected, the pairwise alignment score was selected first and ranked as the most important. Additional attributes of the pairwise similarity, including the number of mismatches and their position, and the number of DNA/RNA bulges were also highly ranked. Additionally, a number of attributes describing the mismatch type (wobble, transversion, purine-purine, and pyrimidine-pyrimidine transitions) were found as important discriminative features. Particularly, we found that the relative frequency of wobble mismatches significantly increases with the total number of mismatches (*p*<0.05; S6 Fig) supporting the notion that wobble mismatches are better tolerated by Cas9 [16].

Extending beyond the pairwise similarity, our results revealed that the types of nucleotides in several positions also affect the sensitivity of CRISPR-Cas9. The selected features indicated the importance of the nucleotide at the second position upstream to the PAM, as was previously observed [28]. Additional nucleotides that were indicated to contribute to the prediction accuracy are the couple of nucleotides at positions 4–5, the site where cleavage occurs, and those in the first five positions downstream to the PAM (S7 Table). In addition, the results pointed at the significance of the nucleotide at the 20^th^ position from the PAM site. Previous studies observed that there is a strong preference for guanine at the 5’-end of the genomic target [56,57]. However, given that all the sgRNAs in our data contain guanine in the 5’-end, the importance of the type of nucleotide at this position could well be an artifact of the assembled dataset.

Among the genomic features that were examined, the presence of the target within DNase I Hypersensitive sites as well as within an exon (either on the coding strand or on the opposite one) were selected. These results support previous observations that reported higher tendency of targets near or around DNase I hypersensitive sites and in coding regions [58–60]. While both attributes signify an exposed DNA structure, the latter is also biased by the selection of on-targets. Interestingly, in addition to a simple categorization of the PAM type (i.e., NGG or NAG), the continuous measure that describes the width of the minor groove surrounding the PAM site was selected. Indeed, some DNA-binding proteins tend to interact with either the minor or major groove of the helix, and it was previously shown that changes in the groove width may affect their fit and therefore their function [61]. Cas9 crystallography highlighted that the PAM-interacting domain of Cas9 makes contacts with the major groove of the PAM duplex [62], and our results suggest that this interaction may be consequently influenced by the groove width.

An additional feature that corroborates the importance of DNA geometry to Cas9 function is DNA enthalpy, which describes the binding affinity of the double helix in and around the genomic site. Our results revealed a symmetric pattern, whereby genomic sites with medium stability are more susceptible to Cas9 cleavage while sites at the extreme ends of the scale are significantly less so (*p* < 0.05 using a permutation test; S7 Fig). This feature, which correlates with other features concerning the local chromatin shape (Fig 4), is indicated to play an important role in predicting Cas9 efficacy. Such geometric features have been previously reported to affect binding of transcription factors and other DNA-binding proteins due to their contribution to the local shape of the double-helix [63,64]. To date, however, the contribution of these aspects to Cas9 affinity has not been explored. We postulate that highly rigid double stranded DNA (dsDNA) with high enthalpy prevents the Cas9 protein from melting the dsDNA and allowing the RNA/DNA duplex to form, while genomic sites with very low enthalpy tend to coil and block access of the enzyme.

### Validation

The learning dataset of CRISTA is based on genome-wide profiling of cleavage intensities of nuclear sites. Thus, targeted evaluation of nuclear sites that were pre-selected according to their similarity to a specified sgRNA could not be integrated within the learning dataset since they would bias the results towards certain features. Yet, those targets could be used as external validation to examine the performance of CRISTA on data that were not used for its training. To this end, datasets of targeted sequencing generated from two studies were examined. Cho et al. [15] analyzed the indel formation of 116 sites by 10 sgRNAs in the human genome using deep sequencing. Similarly, Wang et al. [22] examined 54 sites for two sgRNAs. Combined, these data provided 170 samples of on-targets, off-targets, and uncleaved sites (S2 Text). These datasets differ from the data that were used for the leave-one-sgRNA-out cross-validation procedure in two ways. First, cleavage sites were not detected in an unbiased manner, thus, cleavages of additional potential sites from the reference genome have not been validated and such ones could not be included as a set of uncleaved sites. Second, in contrast to the experimental systems used for our training dataset, the experimental systems used in the studies of Cho et al. and Wang et al. were not sensitive enough to differentiate among nuclear sites that were cleaved at low efficiencies [15,22]. Such sites, which were considered as ‘undetermined’ in the two studies, were marked with zero cleavage intensities for our validation procedure.

Over the sets of 12 sgRNAs and their corresponding targets, CRISTA achieved an averaged Pearson *r*^*2*^ of 0.68, ROC-AUC of 0.7, and PRC-AUC of 0.72 (S9 Table; accuracy measurements of the four alternative tools for each dataset are denoted in S8 and S9 Figs). CRISTA, as well as the other three alternative tools, achieved lower accuracy measurements over the validation data in comparison to the leave-one-sgRNA-out cross-validation procedure. While CRISTA performed better than CCTop and the CFD score according to all three metrics, the averaged Pearson *r*^*2*^ obtained by OptCD (*r*^*2*^ = 0.92) was much higher than those of the other three scoring functions. This could be explained by the dichotomous nature of the OptCD score (see Fig 2, S8 and S9 Figs), which assigns a score of 1.0 to all on-targets and to some sites with a mismatch in unpenalized position, while assigning scores close to 0.0 to nearly all other targets. In contrast, the predictions made by CCTop, the CFD score, and CRISTA produce a more continuous scale. Consequently, assigning the ‘undetermined’ sites with zero cleavage intensities better matches scoring systems that highly penalize off-targets, like OptCD.

## Discussion

CRISTA was developed for the assessment of the cleavage efficacy of a certain genomic target by a specific sgRNA. This assessment integrates two aspects that have been treated separately by currently available tools: those that are designed to predict off-target effects, and those that are aimed at ranking different sgRNAs according to their on-target efficiency. In contrast to the many computational tools that have been developed for these tasks, CRISTA accounts for wider genomic-related attributes in addition to sequence considerations. Additionally, CRISTA considers possible bulges within the DNA site or sgRNA, a concern that was mostly overlooked to date (but see [31,34]).

Our results suggest that bulges are an integral part of the CRISPR system, as they are predicted to occur in approximately 20% of the targets in the evaluated dataset. While a large number of these are targets with low cleavage frequencies, several of them are cleaved at medium-to-high frequencies. These findings are in contrast to the conclusions of Haeussler et al. [27], who argued that bulges are rare and occur in targets that are cleaved at negligible frequencies. This discrepancy could partially be explained if certain combinations of mismatch-gap penalties are assumed when computing the pairwise alignment. While the relative importance of mismatches and bulges to Cas9 activity are underexplored, the experimental results presented by Lin et al. [12], Wang et al. [22], and Ran et al. [20] support our findings that bulges constitute an important component of the off-target spectrum.

We showed that unbiased genome-wide methods for profiling CRISPR target sites generally comply with one another. The discrepancy in the results obtained with Frock data can be explained by the specificities of the HTGTS method [19]. In that study, two alternative approaches were presented: one using sgRNA-generated double strand breaks at on-targets to capture off-targets, and a second approach (termed “universal donor bait HTGTS”) uses known breaks of one sgRNA to capture targets of another. The latter technique was executed on two sgRNAs that were also examined in other studies, and hence belong to the ‘common guides’ set. For these two sgRNAs the predictions made by CRISTA using the leave-study-out procedure were similar to the results obtained for the other studies (Fig 3). In contrast, the sgRNAs that were examined using the first approach were all unique in our dataset. Our analysis demonstrated that the predictions of CRISTA on datasets obtained with this approach were not compatible with the other techniques. Possible explanations to this observation were previously described as bias for sites that are closer in proximity to the on-target [65,66], and we thus chose to eliminate Frock data from the training dataset of CRISTA.

Besides the impact of some known attributes that are important to Cas9 action, namely, attributes that describe pairwise similarity and the nucleotide composition, our results highlight the importance of features that are associated with the DNA geometry, such as the DNA rigidity, double-helix groove width and DNA enthalpy. These attributes are usually used for predicting genomic elements, such as nucleosome organization and transcription factor binding sites, or for determining the optimal setting of empirical procedures (e.g., PCR). Here we found that these features are more influential for predicting CRISPR’s efficacy than measures that are based only on the DNA occupancy. Our findings suggest that integrating local DNA geometry and other genomic features could enhance the prediction and ranking of on-targets. To date, studies that analyzed large datasets of on-targets accounted for position-specific nucleotide identities to evaluate the cleavage efficacy of CRISPR-Cas9 efficacy, and used these to form predictive models [28,56,67–71]. We speculate that incorporating genomic features in the analysis of such data will enhance the ability to rank among on-targets. In addition, we did not find the features concerning the RNA thermodynamics to contribute much to the predictive model. However, the variance of these features in our dataset is low since they are clearly uniform for all samples of the same sgRNA. Possibly their importance will be highlighted when the efficacy of a large number of on-targets is examined.

The CRISTA model described in this study was trained as a regression model, which was fitted to the (transformed) cleavage efficiencies reported in the experimental studies. One difficulty with this approach is the need to combine results from different experimental platforms into a single scale (as described in S1 Text)–a procedure which may bias the results. As an alternative, it is possible to analyze the data within a classification framework. Under such a setting, the data provided by genome-wide profiling of CRISPR-Cas9 could be interpreted as a binary outcome (i.e., all cleaved sites regarded as the set of positives while the uncleaved sites as the negatives). To assess the performance of the learning scheme under these two alternatives (i.e., regression and classification), we implemented a classification model using the Random Forest classification algorithm (S3 Text). Notably, the results obtained using the classification model were very similar—although slightly inferior—to those obtained using the regression model (S10 Fig). This might be expected since the regression model inherently accounts for the differential cleavage propensities among the cleaved sites, whereas the classification approach largely overlooks the complexity present in the experimental data. While it is possible to set a strict threshold on the cleavage propensities above which sites are considered as positives (in contrast to sites that were cleaved at low frequencies and might as well be considered as noise), this setting imposes the difficulty regarding the exact value of the threshold that should be chosen, and raises the question whether such a discretization process extracts the maximum amount of information from the experimental data.

CRISTA was implemented using currently available data, which included published genome-wide profiling of off-targets by CRISPR-Cas9 (the learning dataset) and available predictive tools for feature extraction. The future development of CRISTA would benefit both from the further accumulation of genome-wide profiling of CRISPR-Cas9, as well as from additional features. In turn, an important benefit of CRISTA’s prediction framework is the ability to examine the contribution of various attributes. This use of CRISTA as a platform for hypothesis testing only entails that genome-wide assessment of the examined feature could be provided. A feature that is important for CRISPR-Cas9 mechanism of action would either be highly ranked, or ultimately increase the prediction accuracy.

Genome engineering techniques have evolved rapidly since CRISPR-Cas9 first emerged, introducing alternative endonucleases for manipulating the genome. For example, manipulation of the active domains of the Cas9 enzymes to generate a single-strand break (Cas9-nickase; Cas9n [72,73]) requires targeting of two sites at opposing strands at once, thus yielding a complex with enhanced specificity. Structural biology has been employed to generate Cas9 variants by altering residuals that were identified to mediate the ability of Cas9 to cleave off-target sites, generating eSpCas9 (enhanced SpCas9 [21]) and SpCas9-HF1 (high fidelity SpCas9 [17]). In addition, SpCas9 homologs or other CRISPR endonucleases that differ in their PAM requirements, packaging size, and manner of action, including the Staphylococcus aureus Cas9 (SaCas9 [20]) and the class 2 CRISPR endonuclease, Cpf1 [74], were recently detected, and shown to reduce off-target effect. Nevertheless, Cas9 is still in wide use and protocols that rely on the use of the wild-type SpCas9 for genome engineering, therapeutics, and reverse-genetics have yet to be developed for its alternatives [75–77]. Notably, the learning scheme presented here is not reliant on any specific experimental system, granted this system is not biased towards specific regions of the genome. Thus, future genome-wide experiments can be easily integrated into the learning dataset, including those obtained with Cas9 variants and its orthologs, consequently revealing enzyme-unique characteristics. Taken together, while CRISTA was developed as an inferential tool, such a framework can be further employed to deepen our understanding and to shed light on future research of the CRISPR system.

## Supporting information

S1 Text Data assembly. (PDF)

S2 Text Processing of the data used for validation. (PDF)

S3 Text Converting the regression learning model to classification. (PDF)

S1 Fig Normal distribution of the residuals following a log transformation. Q-Q plot of the genome-wide studies data before and after log transformation (left and right columns), binned to the number of reads reported in: (a) Tsai et al. [16] in U2OS cells, (b) Tsai et al. [16] in HEK293 cell, (c) Slaymaker et al. [21] and Ran et al. [20], (d) Frock et al. [19], and (e) Kleinsteiver et al. [17]. The plots demonstrate that the data distribute similar to a normal distribution after the log transformation.
(PNG)

S2 Fig Cleavage frequencies of the various genome-wide studies compared to GUIDE-Seq. (a) Samples frequencies binned to the number of reads reported in Tsai et al. [16] in U2OS (blue) and HEK293 (green) cell-lines before and after data transformation (left and right). The vertical solid and dashed lines represent the mean number of reads in U2OS and HEK293 cells, respectively. (b-d) comparison of the number of reads reported in Slaymaker et al. [21], Frock et al. [19], and Kleinsteiver et al. [17] to the number of reads reported in Tsai et al. [16] filtered to samples that were found in both. The left column represents the original reported values, whereas the right column represents the transformed values. Pearson *r*^2^ values for each complete set and the mean over the different sgRNAs sets are denoted in the bottom-right corners.
(PNG)

S3 Fig Optimization of pairwise alignment parameters. The colors represent averaged Pearson *r*^*2*^ across the sgRNAs between the pairwise alignment score and the samples cleavage frequencies. For each cell, the optimal pairwise alignment is computed using a match score of 1.0, and the corresponding mismatch and gap penalties.
(PNG)

S4 Fig The effect of allowing for bulges on the number of mismatches. The distribution of the number of mismatches before (blue) and after (light green) allowing for DNA/RNA bulges in the off-targets included in the evaluated positive dataset. The vertical dashed and solid lines represent the mean number of mismatches before and after the alignment, at 3.36 and 3.64, respectively.
(PNG)

S5 Fig CRISTA^+^. The performance of CRISTA^+^ (CRISTA trained on positive samples only) on the positive and negative samples in comparison to the three widely used alternatives. (a) Pearson *r*^*2*^ correlation results in 0.33. (b) Pearson *r*^*2*^ correlation averaged over all the sgRNAs subsets results in 0.63 as opposed to 0.80 received originally. (c-d) The averaged ROC and PRC -AUC values are 0.92 and 0.93 respectively.
(PNG)

S6 Fig The distribution of different types of mismatches as a function of the number of mismatches in the targets of the training dataset. The horizontal axis represents targets with the respective number of mismatches. The vertical axis represents the proportion of mismatches that belong to each type of mismatch (wobble, transversion, transitions of purine-purine, or pyrimidine-pyrimidine) out of the total number of mismatches in the respective group. The impact of wobble substitutions on the cleavage proportion was significantly validated with a chi-square contingency table test, where wobble counts for every bin of mismatches was tested against non-wobble counts (p-value = 0.004).
(PNG)

S7 Fig Effect of the DNA enthalpy on cleavage. Observed cleavage frequency values as a function of DNA enthalpy calculated by the Nearest-Neighbors method [53]. The DNA enthalpy presented here was computed for a 223-nt stretched sequence that includes the 23-nt target, 100 nucleotides downstream, and 100 nucleotides upstream. The two vertical lines represent the 5^th^ and 95^th^ percentiles. The cleavage intensities of nuclear sites with extremely high or low DNA enthalpy were found to be significantly lower than those with medium values (within the 5–95 percentiles), as observed using a permutation test (p-value = 0.021). In this test the DNA enthalpy values were fixed, while the cleavage frequencies were shuffled among the samples of the training set. This procedure was repeated 1000 times. In each iteration, the average cleavage frequency of the samples at the two extreme ends was recorded. The p-value represents the proportion of iterations in which the shuffled average values were lower than the original average.
(PNG)

S8 Fig Accuracy measurements on external datasets. Comparison of the performance of the four computational tools on external data by Cho et al. [15]. Averaged *r*^*2*^, ROC-AUC, PRC-AUC, and Spearman rho coefficient across the sgRNAs are denoted in parenthesis for: (a) CRISTA (0.72, 0.68, 0.72, 0.42), (b) CCTop (0.48, 0.62, 0.67, 0.32), (c) OptCD (0.96, 0.66, 0.7, 0.39), and (d) the CFD score (0.69, 0.65, 0.7, 0.37).
(PNG)

S9 Fig Accuracy measurements on external datasets. Comparison of the performance of the four computational tools on external data by Wang et al. [22]. Averaged *r*^*2*^, ROC-AUC, PRC-AUC, and Spearman rho coefficient across the sgRNAs are denoted in parenthesis for: (a) CRISTA (0.51, 0.81, 0.73, 0.42), (b) CCTop (0.11, 0.77, 0.68, 0.32), (c) OptCD (0.7, 0.81, 0.82, 0.38), and (d) the CFD score (0.37, 0.82, 0.66, 0.44).
(PNG)

S10 Fig Regression versus classification. Comparison of the ROC and PRC curves for the regression and classification models over the assembled dataset. (a) Receiver Operator Characteristics computed over all the samples in the dataset: regression (AUC = 0.96), classification (AUC = 0.95). True positives rate is computed as the true-positives number divided by the number of positives. False-positive rate is computed as the false-positives number divided by the number of negatives. Positives and negatives represent cleaved and uncleaved sites, respectively, in these computations. (b) Precision-Recall curves computed over all the samples in the dataset: regression (AUC = 0.96), classification (AUC = 0.95). Precision is computed as the true-positive number divided by the sum of true-positives and false-positives. Recall is computed as the true-positives number divided by the positives number. (c) Receiver Operator Characteristics curves computed for each sgRNA: regression (averaged AUC = 0.99, *sd* = 0.02), classification (averaged AUC = 0.98, *sd* = 0.03). (d) Precision-Recall curves computed for each sgRNA: regression (averaged AUC = 0.99, *sd* = 0.02), classification (averaged AUC = 0.98, *sd* = 0.03). Mean values are marked with horizontal lines. The whiskers reach 1.5 times past the first and third quartiles.
(PNG)

S1 Table Complete set of sgRNAs used in the training dataset, and the genome-wide studies in which they were profiled. (DOCX)

S2 Table Complete set of samples in dataset before and after pairwise alignment and correction of PAM. The denoted samples compose the complete data before filtration.
(XLSX)

S3 Table Complete set of features used in the learning processes. Features marked with an asterisk were selected first in the Forward Addition procedure (see main text). Features marked with a minus symbol were excluded from the Forward Addition procedure to reduce noise. To calculate features concerning the flanking regions of the target site, e.g., enthalpy, GC content, etc., nucleotide sequences were extracted from the reference genome hg19, using the coordinates provided in the referenced studies [16,17,19–21].
(DOCX)

S4 Table Summary of samples for which the PAM was corrected. Number of targets in each study for which NGG or NAG PAMs were found following the pairwise alignment.
(DOCX)

S5 Table Leave-one-sgRNA-out procedure: Features and predicted scores for the training dataset. All samples in the training dataset (including the negative samples) with detailed features, corrected observed frequencies, CRISTA predictions in the leave-one-sgRNA-out procedure, and CCTop, OptCD, and CFD scores.
(CSV)

S6 Table Averaged Pearson *r*^*2*^ values for reduced sizes of the set of uncleaved sites. The averaged Pearson *r*^*2*^ was re-computed while altering the sample size of this set from 100% to 0% (relative to the size of the set of the cleaved sites).
(DOCX)

S7 Table Leave-study-out procedure: Features and predicted scores for the training dataset. All samples in the training dataset (including the negative samples) with detailed features, corrected observed frequencies, and CRISTA predictions in the leave-study-out procedure.
(CSV)

S8 Table The top 30 selected features in the forward selection procedure. The order of features indicates the selection order. The accuracy measurements (RMSE, Pearson *r*^*2*^, ROC AUC, and PRC AUC) in each row are computed over the model when trained on the incremental set of features that were selected until that point.
(DOCX)

S9 Table Validation dataset: Features and predicted scores. All samples in the validation dataset with detailed features, corrected observed frequencies, CRISTA predictions, and CCTop, OptCD, and CFD scores.
(CSV)
