# Supplementary material for: A machine learning approach for predicting CRISPR-Cas9 cleavage efficiencies and patterns underlying its mechanism of action
Source: PLoS Comput Biol. 2017 Oct 16;13(10):e1005807. doi: 10.1371/journal.pcbi.1005807 (PMC5658169; doi:10.1371/journal.pcbi.1005807)
Supplement: S1 Text — (PDF) [file pcbi.1005807.s001.pdf]

S1 Text: Data assembly

GUIDE-seq data (Tsai et al. [16], Kleinstiver et al. [17])

Tsai et al. carried out unbiased off-target detection using GUIDE-seq for 10 different 20-nt sgRNAs. Six sgRNAs were applied to endogenous human genes in U2OS cell-line and four to HEK293 cell-line. The platform for HEK293 cells experiments was different in experimental conditions from that of the U2OS cell-line, therefore, we scaled their intensities by multiplying the number of reads by a factor, which is the ratio between the average on-targets read counts above the fifth percentile in U2OS cells and that of the HEK293 cells (S1a Figure).

Kleinsteiver et al. carried out GUIDE-seq for ten sgRNAs in U2OS cell-line, four of which were used in Tsai et al. Because there might be differences in the reported number of reads due to different experimental conditions, we considered the two sources of data to be distinct.

Some sites in Tsai and Kleinsteiver data follow a non-canonical PAM even if one could be found 2-nt upstream or downstream. These cases were probably missed since the studies by Tsai et al. [1] and Kleinsteiver et al. [2] did not allow for the occurrences of DNA/RNA bulges. To correct such instances, we re-evaluated the localization of all non-NGG sites. Following the introduction of gaps in the alignment, the location of 49 and 17 instances of Tsai and Kleinsteiver data, respectively, were shifted by one or two nucleotides such that NGG or NAG PAMs were found (S4 Table).

BLESS data (Ran et al. [20], Slaymaker et al. [21])

Ran et al. use the BLESS technique to detects genomic double-strand breaks at CRISPR transfected HEK293 cells. Ran data contain two 20-nt sgRNAs for targeting two loci in the human EMX1 gene, introduced to HEK293 cell-line by SpCas9 enzymes.

Slaymaker et al. used BLESS to compare SpCas9 to a newly engineered nuclease. It was targeted at sites of EMX-1 and VEGFA human genes. Both studies were conducted under identical experimental settings and were thus united.

HTGTS data (Frock et al. [19])

The HTGTS (high-throughput genome-wide translocation sequencing) method performed by Frock et al. exploits CRISPR-SpCas9 on-targets to detect the corresponding off-targets by profiling translocations. HEK293 cells were transfected with wild-type SpCas9 and 9 different 20-nt long sgRNAs. Seven sgRNAs on-targets served as a 'bait', hence the cleavage intensity of the on-target site is unknown. Off-targets of VEGFA and EMX1 genes targets were detected using a 'universal' bait. Similar to Tsai data, the lists of detected cleaved sites are denoted by their genomic coordinates in the genome assembly GrCh37 (173 altogether). Because Frock et al. [5] did not allow for the occurrences of DNA/RNA bulges, we re-evaluated the PAM location and corrected 22 instances (S4 Table).

Data scaling

In order to merge the samples into a single dataset, Kleinsteiver, Frock, Ran, and Slaymaker data were scaled to Tsai data (which is the most inclusive study) as follows: first, since the data is highly right-skewed, we applied log transformation to enforce normally distributed residuals (S1 Fig). Then, for each study, we kept only the targets that are shared with Tsai data (termed 'common guides'; S1 Table). Next, we fitted the cleavage frequencies of these targets between the two studies with linear regression (S2 Fig), and used the inferred parameters to transform the rest of the targets.
