# Supplementary material for: A machine learning approach for predicting CRISPR-Cas9 cleavage efficiencies and patterns underlying its mechanism of action
Source: PLoS Comput Biol. 2017 Oct 16;13(10):e1005807. doi: 10.1371/journal.pcbi.1005807 (PMC5658169; doi:10.1371/journal.pcbi.1005807)
Supplement: S2 Text — (PDF) [file pcbi.1005807.s002.pdf]

S2 Text: Processing of the data used for validation.

Cho et al. [15]

Cho et al. used deep sequencing to assess the cleavage efficacies of 116 genomic sites that could potentially be cleaved by ten sgRNAs. These sites were selected such that they retain up to three mismatches from their corresponding sgRNA.

Wang et al. [22]

Wang et al. used deep sequencing to assess the cleavage efficacies of 54 genomic sites that could potentially be cleaved by two sgRNAs. These sites were selected such that there could be up to one mismatch at the first ten PAM-proximal nucleotides, and no more than four mismatches overall.

External data transformation

Similar to the data in our training dataset, these data represent indel formation frequencies. Therefore, we applied log transformation to them. To represent the data (S8-S9 Figs), we divided both datasets by their maximal values accordingly.
