# Supplementary material for: A machine learning approach for predicting CRISPR-Cas9 cleavage efficiencies and patterns underlying its mechanism of action
Source: PLoS Comput Biol. 2017 Oct 16;13(10):e1005807. doi: 10.1371/journal.pcbi.1005807 (PMC5658169; doi:10.1371/journal.pcbi.1005807)
Supplement: S3 Text — (PDF) [file pcbi.1005807.s003.pdf]

S3 Text: Converting the regression learning model to classification.

Methods

To assess the performance of CRISTA as a classifier rather than a regression model, the cleaved sites were labeled as positives and the uncleaved sites as negatives. The learning scheme was performed using the RandomForestClassifier algorithm, implemented in the python scikit-learn module [1,2]. The data processing and performance evaluation were done in a leave-one-sgRNA-out cross-validation procedure as explained for the regression model. The set of features was identical to that used for the regression model (S3 Table). ROC-AUC and PRC-AUC were computed over the whole data, as well as averaged over the sets of sgRNAs. Our results demonstrate that the AUC values obtained by the classifier are highly similar, yet slightly inferior, to those obtained using the regression models (S10 Fig).

---

1. Breiman L. Random forests. In: Flach PA, editor. Machine Learning. 0885–6125th ed. New-York: Springer US; 2001. pp. 1–35. doi:10.1023/A:1010933404324

2. Pedregosa FABIANPEDREGOSA F, Alexandre Gramfort N, Michel V, Thirion BERTRANDTHIRION B, Grisel O, Blondel M, et al. Scikit-learn: Machine Learning in Python Gaël Varoquaux. J Mach Learn Res. 2011;12:2825–2830. Available: http://www.jmlr.org/papers/volume12/pedregosa11a/pedregosa11a.pdf
