# Supplementary figures and images for: A machine learning approach for predicting CRISPR-Cas9 cleavage efficiencies and patterns underlying its mechanism of action
Source: PLoS Comput Biol. 2017 Oct 16;13(10):e1005807. doi: 10.1371/journal.pcbi.1005807 (PMC5658169; doi:10.1371/journal.pcbi.1005807)

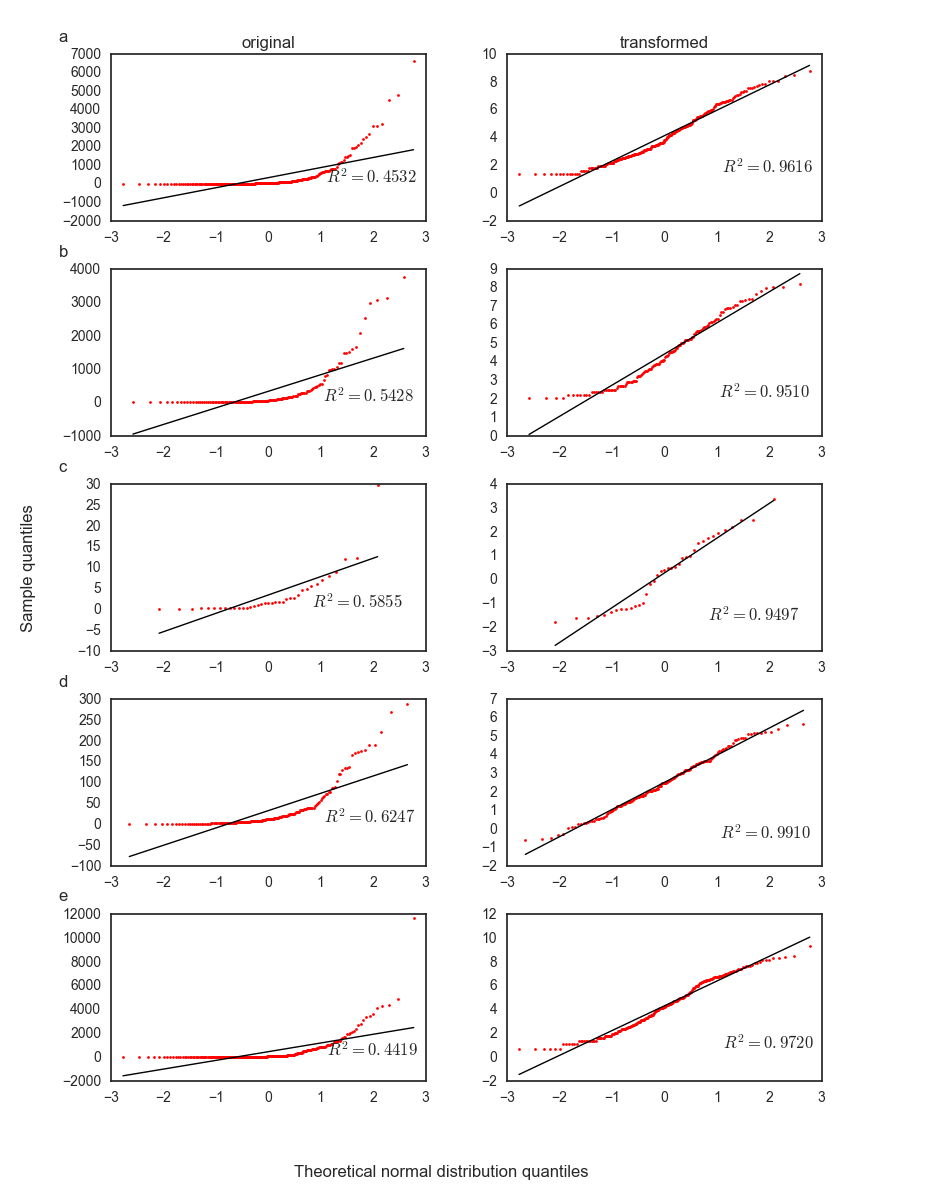

Supplement: S1 Fig — Q-Q plot of the genome-wide studies data before and after log transformation (left and right columns), binned to the number of reads reported in: (a) Tsai et al. [16] in U2OS cells, (b) Tsai et al. [16] in HEK293 cell, (c) Slaymaker et al. [21] and Ran et al. [20], (d) Frock et al. [19], and (e) Kleinsteiver et al. [17]. The plots demonstrate that the data distribute similar to a normal distribution after the log transformation. (PNG) [file pcbi.1005807.s004.png]

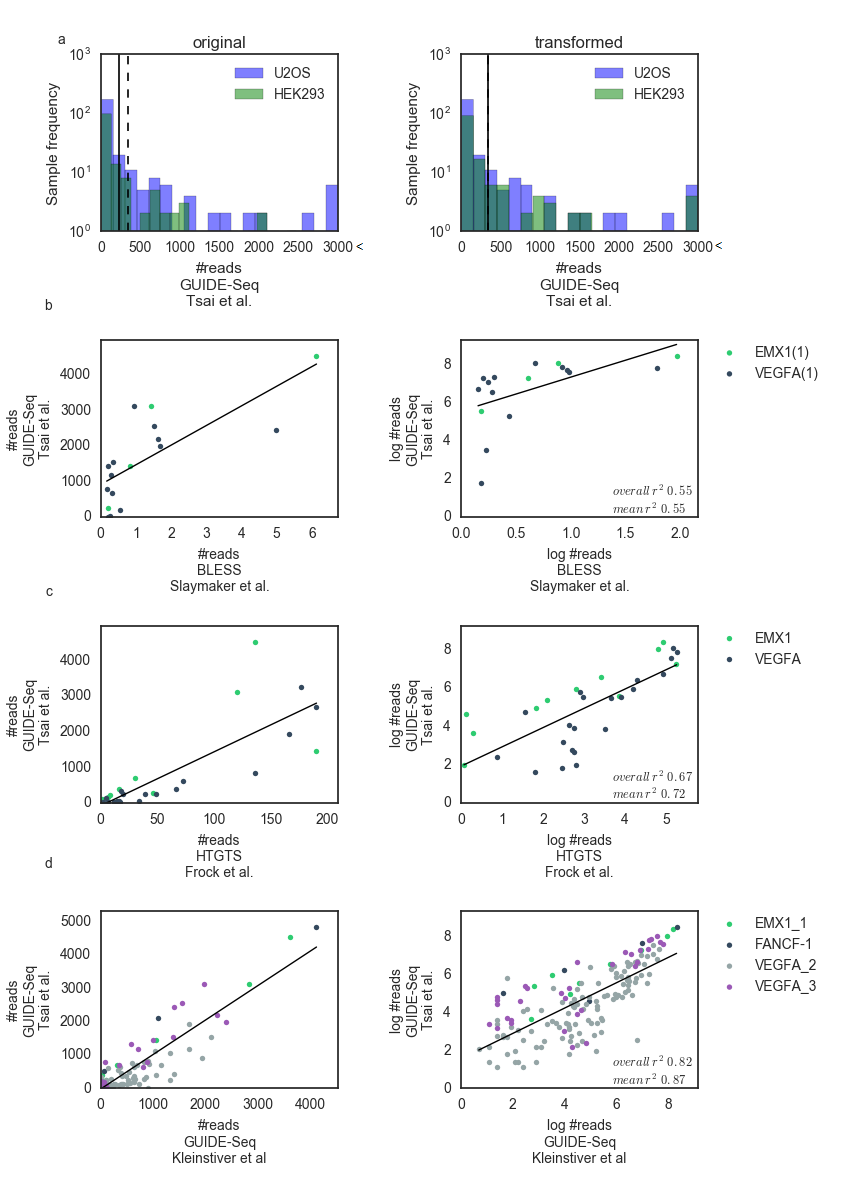

Supplement: S2 Fig — (a) Samples frequencies binned to the number of reads reported in Tsai et al. [16] in U2OS (blue) and HEK293 (green) cell-lines before and after data transformation (left and right). The vertical solid and dashed lines represent the mean number of reads in U2OS and HEK293 cells, respectively. (b-d) comparison of the number of reads reported in Slaymaker et al. [21], Frock et al. [19], and Kleinsteiver et al. [17] to the number of reads reported in Tsai et al. [16] filtered to samples that were found in both. The left column represents the original reported values, whereas the right column represents the transformed values. Pearson r2 values for each complete set and the mean over the different sgRNAs sets are denoted in the bottom-right corners. (PNG) [file pcbi.1005807.s005.png]

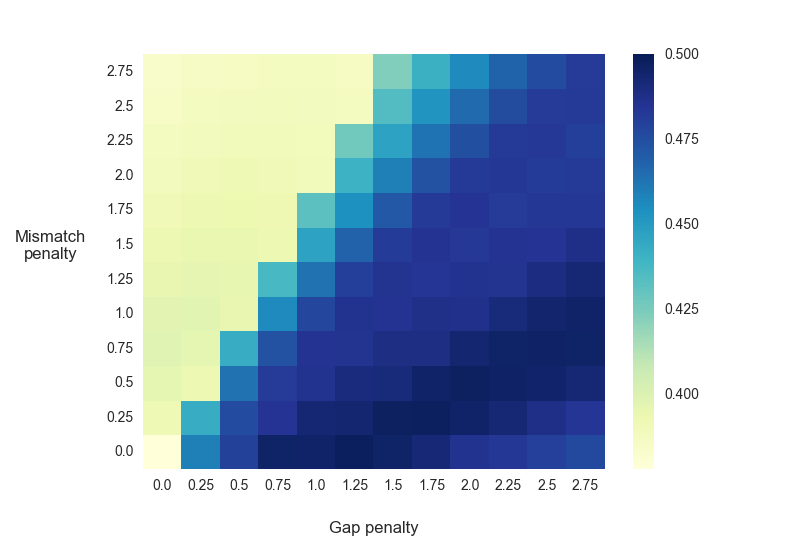

Supplement: S3 Fig — The colors represent averaged Pearson r2 across the sgRNAs between the pairwise alignment score and the samples cleavage frequencies. For each cell, the optimal pairwise alignment is computed using a match score of 1.0, and the corresponding mismatch and gap penalties. (PNG) [file pcbi.1005807.s006.png]

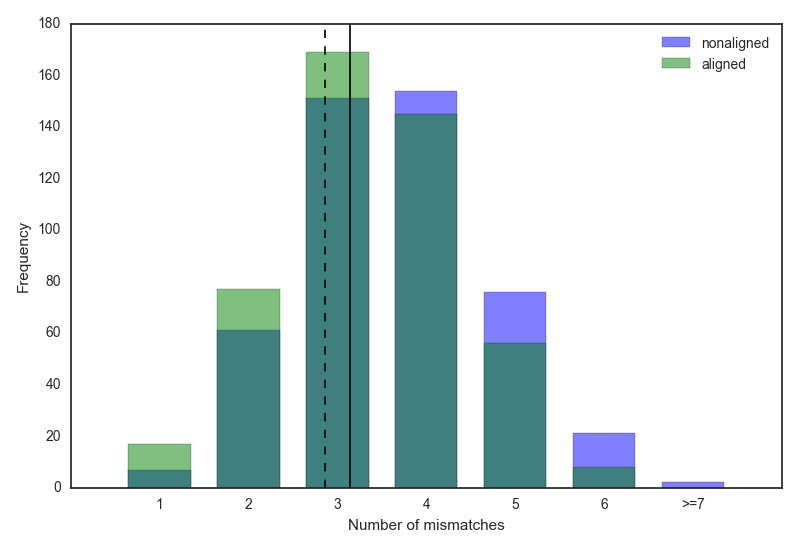

Supplement: S4 Fig — The distribution of the number of mismatches before (blue) and after (light green) allowing for DNA/RNA bulges in the off-targets included in the evaluated positive dataset. The vertical dashed and solid lines represent the mean number of mismatches before and after the alignment, at 3.36 and 3.64, respectively. (PNG) [file pcbi.1005807.s007.png]

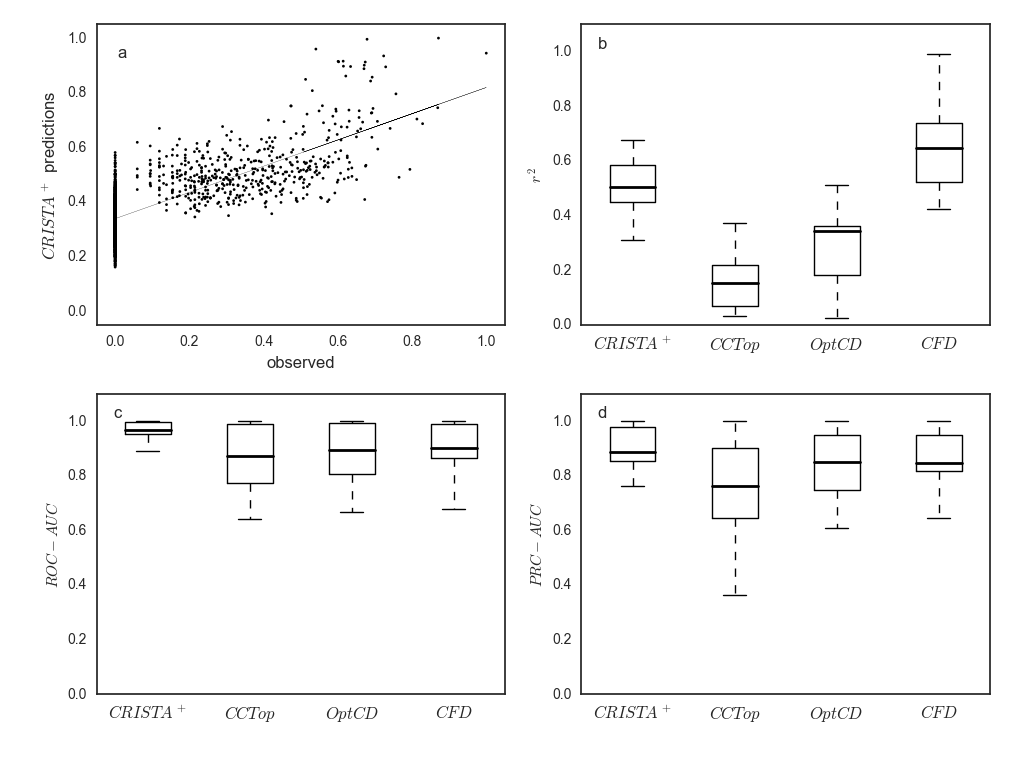

Supplement: S5 Fig — The performance of CRISTA+ (CRISTA trained on positive samples only) on the positive and negative samples in comparison to the three widely used alternatives. (a) Pearson r2 correlation results in 0.33. (b) Pearson r2 correlation averaged over all the sgRNAs subsets results in 0.63 as opposed to 0.80 received originally. (c-d) The averaged ROC and PRC -AUC values are 0.92 and 0.93 respectively. (PNG) [file pcbi.1005807.s008.png]

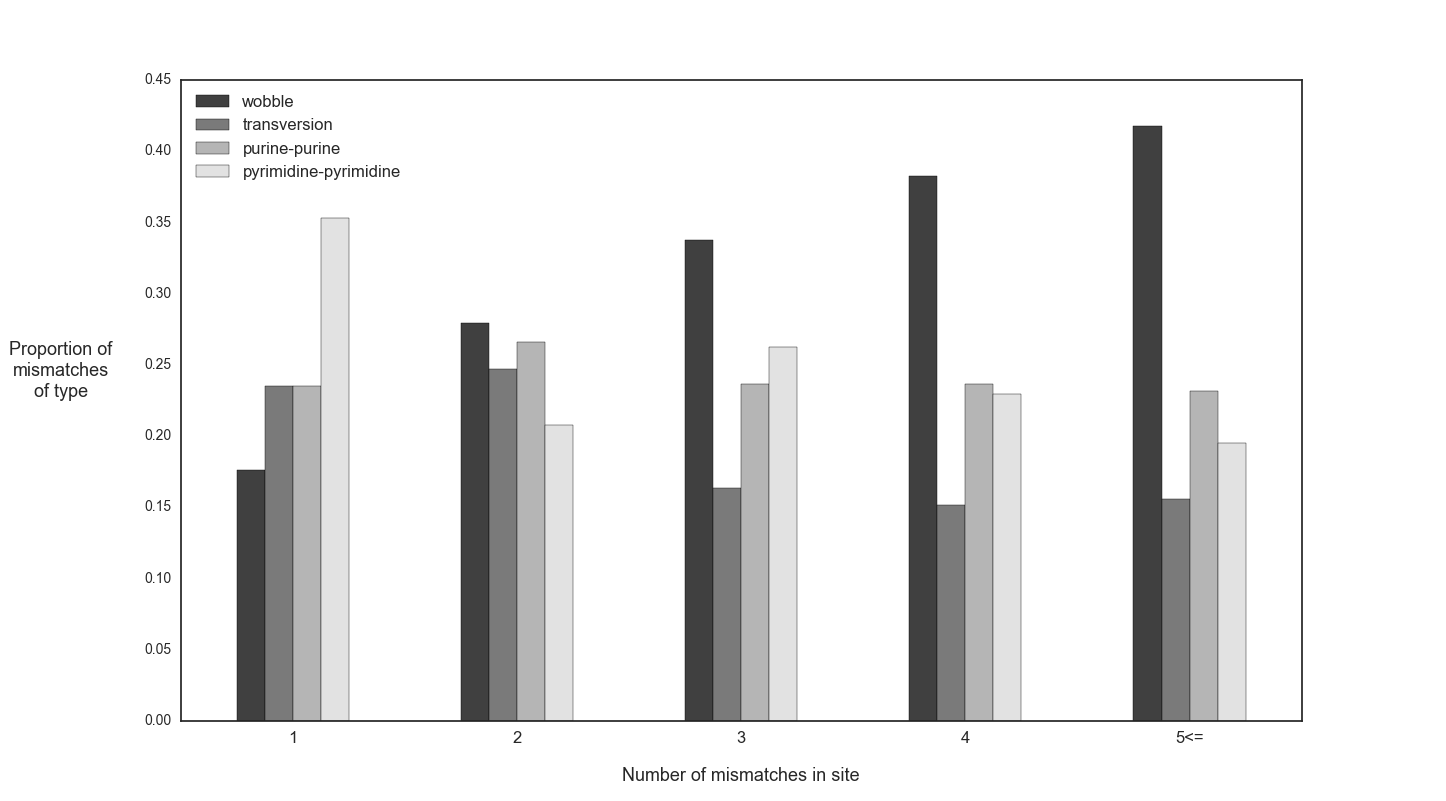

Supplement: S6 Fig — The horizontal axis represents targets with the respective number of mismatches. The vertical axis represents the proportion of mismatches that belong to each type of mismatch (wobble, transversion, transitions of purine-purine, or pyrimidine-pyrimidine) out of the total number of mismatches in the respective group. The impact of wobble substitutions on the cleavage proportion was significantly validated with a chi-square contingency table test, where wobble counts for every bin of mismatches was tested against non-wobble counts (p-value = 0.004). (PNG) [file pcbi.1005807.s009.png]

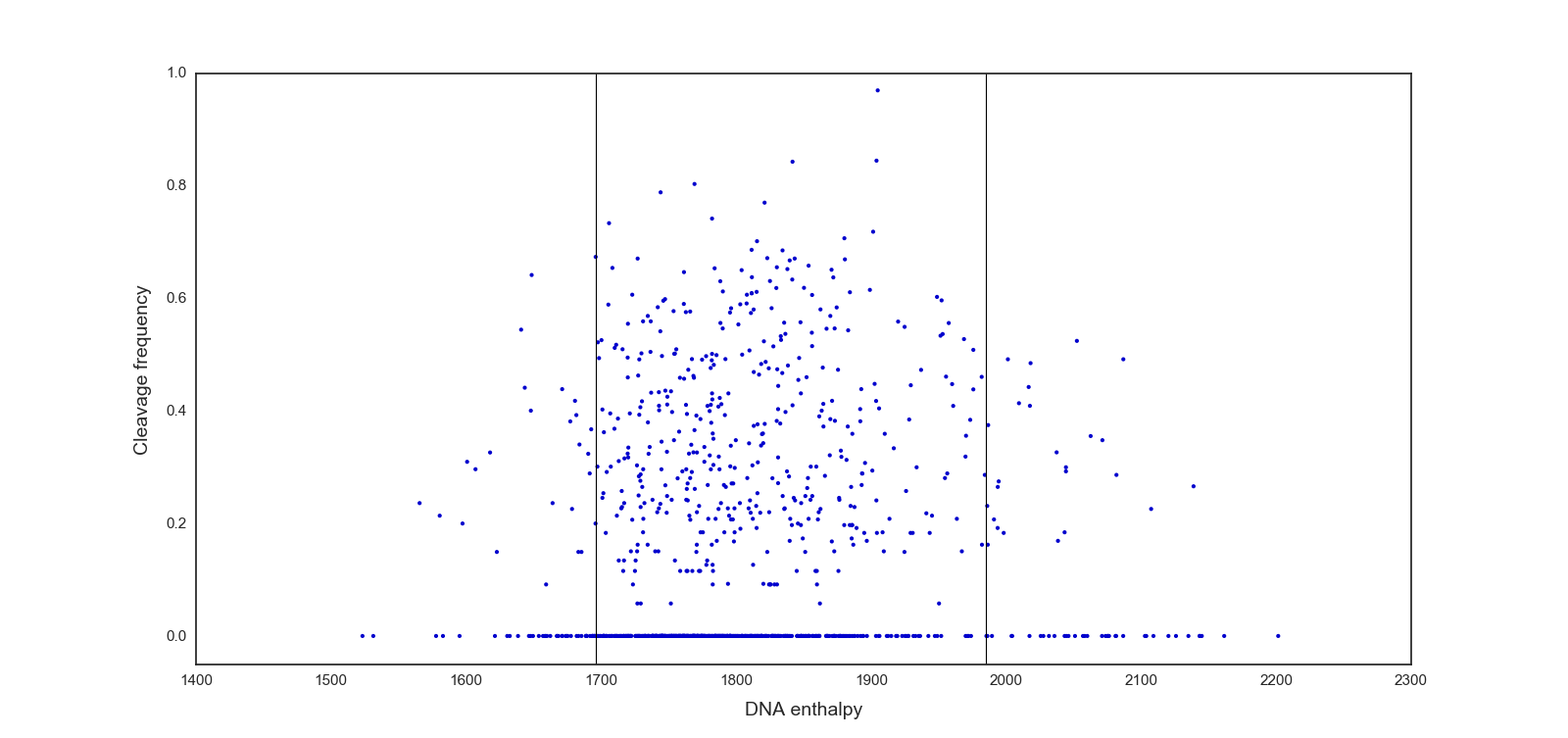

Supplement: S7 Fig — Observed cleavage frequency values as a function of DNA enthalpy calculated by the Nearest-Neighbors method [53]. The DNA enthalpy presented here was computed for a 223-nt stretched sequence that includes the 23-nt target, 100 nucleotides downstream, and 100 nucleotides upstream. The two vertical lines represent the 5th and 95th percentiles. The cleavage intensities of nuclear sites with extremely high or low DNA enthalpy were found to be significantly lower than those with medium values (within the 5–95 percentiles), as observed using a permutation test (p-value = 0.021). In this test the DNA enthalpy values were fixed, while the cleavage frequencies were shuffled among the samples of the training set. This procedure was repeated 1000 times. In each iteration, the average cleavage frequency of the samples at the two extreme ends was recorded. The p-value represents the proportion of iterations in which the shuffled average values were lower than the original average. (PNG) [file pcbi.1005807.s010.png]

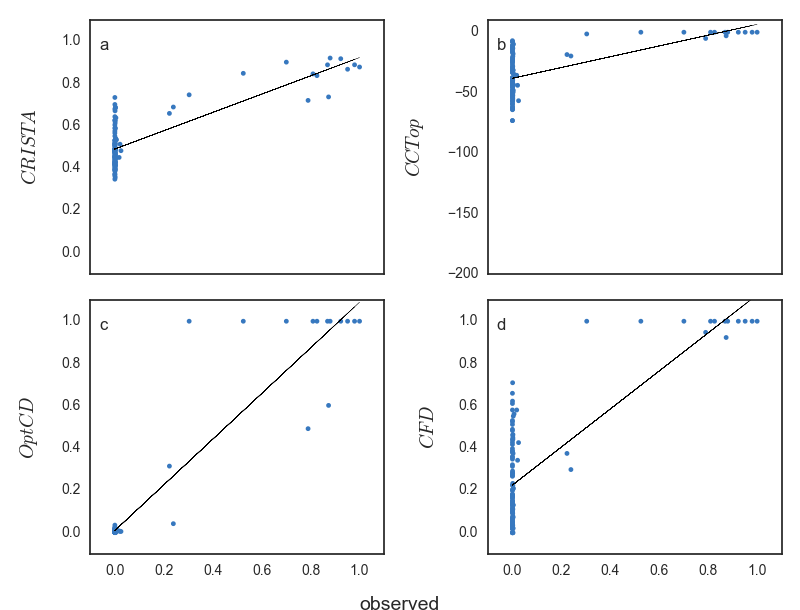

Supplement: S8 Fig — Comparison of the performance of the four computational tools on external data by Cho et al. [15]. Averaged r2, ROC-AUC, PRC-AUC, and Spearman rho coefficient across the sgRNAs are denoted in parenthesis for: (a) CRISTA (0.72, 0.68, 0.72, 0.42), (b) CCTop (0.48, 0.62, 0.67, 0.32), (c) OptCD (0.96, 0.66, 0.7, 0.39), and (d) the CFD score (0.69, 0.65, 0.7, 0.37). (PNG) [file pcbi.1005807.s011.png]

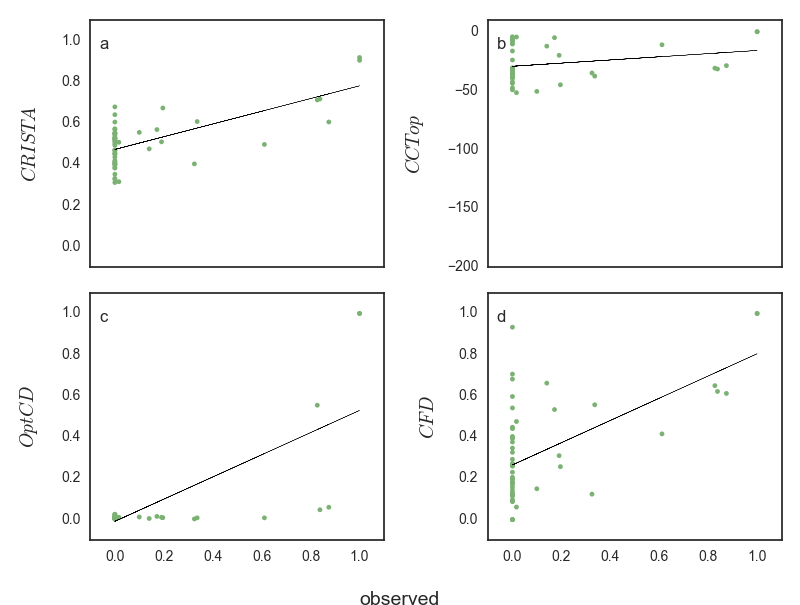

Supplement: S9 Fig — Comparison of the performance of the four computational tools on external data by Wang et al. [22]. Averaged r2, ROC-AUC, PRC-AUC, and Spearman rho coefficient across the sgRNAs are denoted in parenthesis for: (a) CRISTA (0.51, 0.81, 0.73, 0.42), (b) CCTop (0.11, 0.77, 0.68, 0.32), (c) OptCD (0.7, 0.81, 0.82, 0.38), and (d) the CFD score (0.37, 0.82, 0.66, 0.44). (PNG) [file pcbi.1005807.s012.png]

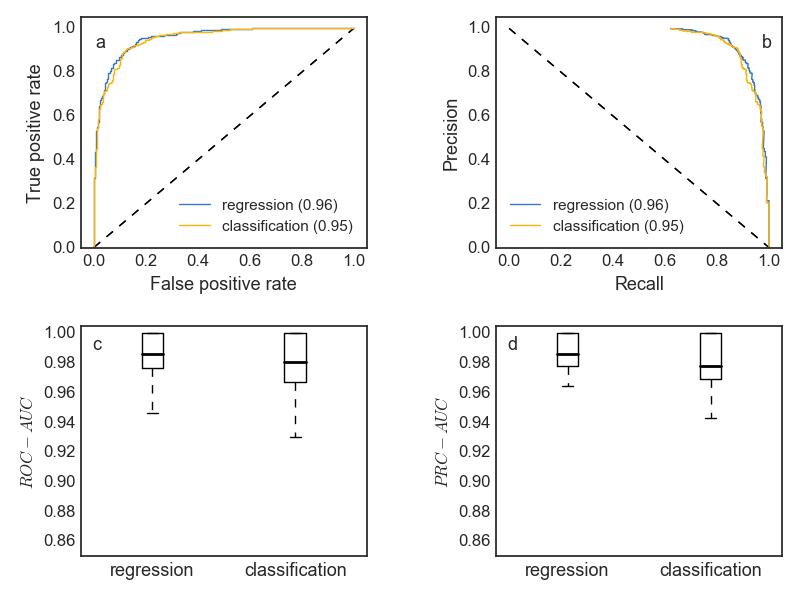

Supplement: S10 Fig — Comparison of the ROC and PRC curves for the regression and classification models over the assembled dataset. (a) Receiver Operator Characteristics computed over all the samples in the dataset: regression (AUC = 0.96), classification (AUC = 0.95). True positives rate is computed as the true-positives number divided by the number of positives. False-positive rate is computed as the false-positives number divided by the number of negatives. Positives and negatives represent cleaved and uncleaved sites, respectively, in these computations. (b) Precision-Recall curves computed over all the samples in the dataset: regression (AUC = 0.96), classification (AUC = 0.95). Precision is computed as the true-positive number divided by the sum of true-positives and false-positives. Recall is computed as the true-positives number divided by the positives number. (c) Receiver Operator Characteristics curves computed for each sgRNA: regression (averaged AUC = 0.99, sd = 0.02), classification (averaged AUC = 0.98, sd = 0.03). (d) Precision-Recall curves computed for each sgRNA: regression (averaged AUC = 0.99, sd = 0.02), classification (averaged AUC = 0.98, sd = 0.03). Mean values are marked with horizontal lines. The whiskers reach 1.5 times past the first and third quartiles. (PNG) [file pcbi.1005807.s013.png]
