# Supplementary material for: A machine learning approach for predicting CRISPR-Cas9 cleavage efficiencies and patterns underlying its mechanism of action
Source: PLoS Comput Biol. 2017 Oct 16;13(10):e1005807. doi: 10.1371/journal.pcbi.1005807 (PMC5658169; doi:10.1371/journal.pcbi.1005807)
Supplement: S1 Table — (DOCX) [file pcbi.1005807.s014.docx]

**S1 Table. Complete set of sgRNAs used in the training dataset, and the genome-wide studies in which they were profiled.**

| sgRNA sequence | Profiling method |  |
| --- | --- | --- |
| GAGTCCGAGCAGAAGAAGAA | GUIDE-seq [1], GUIDE-seq [2], BLESS [4], HTGTS [5] | *Common guides* |
| GGAATCCCTTCTGCAGCACC | GUIDE-seq [1], GUIDE-seq [2] |  |
| GGGTGGGGGGAGTTTGCTCC | GUIDE-seq [1], HTGTS [5] |  |
| GACCCCCTCCACCCCGCCTC | GUIDE-seq [1], GUIDE-seq [2] |  |
| GGTGAGTGAGTGTGTGCGTG | GUIDE-seq [1], GUIDE-seq [2], BLESS [4] |  |
| GTCATCTTAGTCATTACCTG | GUIDE-seq [1] | *Unique guides* |
| GGGAAAGACCCAGCATCCGT | GUIDE-seq [1] |  |
| GAACACAAAGCATAGACTGC | GUIDE-seq [1] |  |
| GGCCCAGACTGAGCACGTGA | GUIDE-seq [1] |  |
| GGCACTGCGGCTGGAGGTGG | GUIDE-seq [1] |  |
| GTCACCTCCAATGACTAGGG | GUIDE-seq [2] |  |
| GCTGCAGAAGGGATTCCATG | GUIDE-seq [2] |  |
| GGCGGCTGCACAACCAGTGG | GUIDE-seq [2] |  |
| GCTCCAGAGCCGTGCGAATG | GUIDE-seq [2] |  |
| GCATTTTCAGGAGGAAGCGA | GUIDE-seq [2] |  |
| GTGCGGCAAGAGCTTCAGCC | GUIDE-seq [2] |  |
| GCCTCCCCAAAGCCTGGCCA | BLESS [3] |  |
| GGCCAGGCTTTGGGGAGGCC | BLESS [3] |  |
| ACCCCTTCCCCACCTACCTT | HTGTS [5] |  |
| GACCTTAAGGTTTTTGTGGA | HTGTS [5] |  |
| GACTTGTTTTCATTGTTCTC | HTGTS [5] |  |
| GCACCTAACATGATATATTA | HTGTS [5] |  |
| GCCTCTCCCCACCCACCCTT | HTGTS [5] |  |
| GCCTCTTTCCCACCCACCTT | HTGTS [5] |  |
| TCCTCCTCCCCACCCACCTT | HTGTS [5] |  |
