# Supplementary material for: A machine learning approach for predicting CRISPR-Cas9 cleavage efficiencies and patterns underlying its mechanism of action
Source: PLoS Comput Biol. 2017 Oct 16;13(10):e1005807. doi: 10.1371/journal.pcbi.1005807 (PMC5658169; doi:10.1371/journal.pcbi.1005807)
Supplement: S3 Table — Features marked with an asterisk were selected first in the Forward Addition procedure (see main text). Features marked with a minus symbol were excluded from the Forward Addition procedure to reduce noise. To calculate features concerning the flanking regions of the target site, e.g., enthalpy, GC content, etc., nucleotide sequences were extracted from the reference genome hg19, using the coordinates provided in the referenced studies [16,17,19–21]. (DOCX) [file pcbi.1005807.s016.docx]

**S3 Table. Complete set of features used in the learning processes.** Features marked with an asterisk were selected first in the Forward Addition procedure (see main text). Features marked with a minus symbol were excluded from the Forward Addition procedure to reduce noise. To calculate features concerning the flanking regions of the target site, e.g., enthalpy, GC content, etc., nucleotide sequences were extracted from the reference genome hg19, using the coordinates provided in the referenced studies [1–5].

| Feature | Remarks |
| --- | --- |
| Features derived from pairwise sequence alignment | |
| pairwise alignment score | See Methods. |
| number of total bulges^-^ |  |
| number of RNA bulges* |  |
| number of DNA bulges* |  |
| number of mismatches* |  |
| number of linked mismatches and bulges |  |
| linked mismatches and bulges average length |  |
| number of mismatches at positions 1-4 |  |
| number of mismatches at positions 5-8 |  |
| number of mismatches at positions 9-12 |  |
| number of mismatches at positions 13-16 |  |
| number of mismatches at positions 17-20* |  |
| number of pyrimidine-pyrimidine mismatches |  |
| number of purine-purine mismatches* |  |
| number of wobble mismatches (rU-dG or rG-dT) * |  |
| number of other transversion mismatches* |  |
| Nucleotide identities | |
| PAM type* |  |
| type of nucleotide at PAM N site |  |
| type of nucleotide at 5'-end * |  |
| nucleotides at positions 1-2 upstream to PAM site |  |
| nucleotides at positions 2-3 upstream to PAM site |  |
| nucleotides at positions 3-4 upstream to PAM site |  |
| nucleotides at positions 4-5 upstream to PAM site |  |
| nucleotides at position 1 downstream to PAM |  |
| nucleotides at position 2 downstream to PAM |  |
| nucleotides at position 3 downstream to PAM |  |
| nucleotides at position 4 downstream to PAM |  |
| nucleotides at position 5 downstream to PAM |  |
| GC content at 73-nt upstream and including the nuclear site |  |
| GC content at 73-nt downstream and including the nuclear site |  |
| GC content at the 23-nt and along an extended region of 123-nt (the nuclear target and 50-nt from each side) |  |
| Adenine occupancy in 20-nt nuclear site |  |
| Cytosine occupancy in 20-nt nuclear site |  |
| Guanine occupancy in 20-nt nuclear site |  |
| Thymine occupancy in 20-nt nuclear site |  |
| sgRNA secondary structure features | |
| sgRNA 20-nt minimum free energy | The Vienna RNAfold [6] package was used to obtain the minimum free energy structure for both the 20-nt base pairing region of the sgRNA and the whole sgRNA sequence [7] (including 102 nucleotides). |
| sgRNA 20-nt ensemble minimum free energy |  |
| sgRNA 20-nt minimum free energy frequency |  |
| sgRNA 20-nt ensemble diversity |  |
| number of sgRNA secondary structure paired nucleotides |  |
| whole sgRNA minimum free energy |  |
| whole sgRNA ensemble minimum free energy |  |
| whole sgRNA minimum free energy frequency |  |
| whole sgRNA ensemble diversity |  |
| Genomic location | |
| chromosome number |  |
| distance from centromere | The coordinates of telomeres and centromeres were obtained from the relevant UCSC genome assembly [8]. |
| distance from telomere |  |
| Features from experimental databases | |
| DNAse I Hypersensitive Site (DHS) signal value within the nuclear site* | Retrieved from cell-line specific databases in the ENCODE project at UCSC [9]. Since there is a high correlation between DHS mapping in different human cell-lines [10], the missing U2OS cell data was replaced with the non-cancerous cell-line of Osteoblasts. |
| distance from closest DHS |  |
| nucleosome occupancy in the nuclear site | Retrieved from control data of nucleosome mapping done by micrococcal nuclease digestion by [LeRoy et al. ,11]. The published genomic coordinates are denoted by Grch38/hg16 annotations, and converted to hg19 with liftOver [12] |
| distance from closest nucleosome |  |
| genomic subcompartment of the nuclear site | Retrieved from data of in situ Hi-C by [Rao et al. ,13] |
| gene expression level at nuclear site (PAM strand) | Data for U2OS cell-line were retrieved from control data done by [Ajiro et al. ,14] and parsed using BLAT [15] with dedicated python scripts. The data for K562 and HEK293 cells was retrieved from the ENCODE project at UCSC [9] |
| gene expression level at nuclear site (non-PAM strand) |  |
| A Boolean attribute indicating whether the nuclear site is located within an exon (PAM strand) |  |
| A Boolean attribute indicating whether the nuclear site is located within an exon (non-PAM strand) * |  |
| A Boolean attribute indicating whether the nuclear site is located in transcription region |  |
| A Boolean attribute indicating whether the nuclear site is located in coding region |  |
| DNA enthalpy and geometry features | |
| DNA enthalpy of the 23-nt nuclear site | DNA enthalpy between the double-helix strands at the nuclear target was calculated using the nearest-neighbor pairwise interactions method described by Breslauer [16]. The enthalpy was assessed along the 23-bp genomic site and along an extended region of 123-nt (the nuclear target and 50-nt from each side). |
| DNA enthalpy at the 23-nt and along an extended region of 123-nt (the nuclear target and 50-nt from each side)* |  |
| average HelT of the 23-nt nuclear site | Local DNA geometry features were calculated using DNAshape [17], which estimates four parameters given a nucleic sequence: Propeller-Twist (ProT) defines the rotation of one base with respect to the other in the same base pair; Helix-Twist (HelT) represents the angle of rotation in the helix between two consecutive base-pairs; Roll defines the rotation around the slide axis ; Minor Groove Width (MGW) defines the distance between the two pairing strands in the DNA double helix. Based on preliminary trials, we used a total of eight features: the minimum MGW and the means of the ProT, HelT, and Roll throughout the 23-nt nuclear site sequence, and their values exclusively around the PAM (considering the NNGGN sequences for MGW and ProT and NNGGNN sequence for HelT and Roll). |
| average Roll of the 23-nt nuclear site |  |
| average ProT of the 23-nt nuclear site |  |
| MGW at PAM site given the NNGGN sequence* |  |
| HelT at PAM site given the NNGGN sequence |  |
| Roll at PAM site given the NNGGNN sequence |  |
| ProT at PAM site given the NNGGNN sequence |  |
| minimum MGW within the 23-nt nuclear site |  |

**References:**

1. Tsai SQ, Zheng Z, Nguyen NT, Liebers M, Topkar V V, Thapar V, et al. GUIDE-seq enables genome-wide profiling of off-target cleavage by CRISPR-Cas nucleases. Nat Biotechnol. 2014;33: 187–197. doi:10.1038/nbt.3117

2. Frock RL, Hu J, Meyers RM, Ho Y, Kii E, Alt FW. Genome-wide detection of DNA double-stranded breaks induced by engineered nucleases. Nat Biotechnol. 2014;33: 179–186. doi:10.1038/nbt.3101

3. Ran FA, Cong L, Yan WX, Scott D a., Gootenberg JS, Kriz AJ, et al. In vivo genome editing using Staphylococcus aureus Cas9. Nature. 2015;520: 186–190. doi:10.1038/nature14299

4. Slaymaker IM, Gao L, Zetsche B, Scott DA, Yan WX, Zhang F. Rationally engineered Cas9 nucleases with improved specificity. Science (80- ). 2015; doi:10.1126/science.aad5227

5. Kleinstiver BP, Prew MS, Tsai SQ, Topkar V V., Nguyen NT, Zheng Z, et al. Engineered CRISPR-Cas9 nucleases with altered PAM specificities. Nature. 2015;523: 481–5. doi:10.1038/nature14592

6. Lorenz R, Bernhart SH, Höner Zu Siederdissen C, Tafer H, Flamm C, Stadler PF, et al. ViennaRNA Package 2.0. Algorithms Mol Biol. 2011;6: 26. doi:10.1186/1748-7188-6-26

7. Anders C, Niewoehner O, Duerst A, Jinek M. Structural basis of PAM-dependent target DNA recognition by the Cas9 endonuclease. Nature. Nature Publishing Group; 2014;513: 569–573. doi:10.1038/nature13579

8. Rosenbloom KR, Armstrong J, Barber GP, Casper J, Clawson H, Diekhans M, et al. The UCSC Genome Browser database: 2015 update. Nucleic Acids Res. 2014;43: D670-681. doi:10.1093/nar/gku1177

9. Rosenbloom KR, Sloan CA, Malladi VS, Dreszer TR, Learned K, Kirkup VM, et al. ENCODE data in the UCSC Genome Browser: year 5 update. Nucleic Acids Res. 2013;41: D56-63. doi:10.1093/nar/gks1172

10. Crawford GE, Holt IE, Whittle J, Webb BD, Tai D, Davis S, et al. Genome-wide mapping of DNase hypersensitive sites using massively parallel signature sequencing (MPSS). Genome Res. Cold Spring Harbor Laboratory Press; 2006;16: 123–31. doi:10.1101/gr.4074106

11. LeRoy G, Chepelev I, DiMaggio PA, Blanco MA, Zee BM, Zhao K, et al. Proteogenomic characterization and mapping of nucleosomes decoded by Brd and HP1 proteins. Genome Biol. BioMed Central; 2012;13: R68. doi:10.1186/gb-2012-13-8-r68

12. Hinrichs AS, Karolchik D, Baertsch R, Barber GP, Bejerano G, Clawson H, et al. The UCSC Genome Browser Database: update 2006. Nucleic Acids Res. Oxford University Press; 2006;34: D590-8. doi:10.1093/nar/gkj144

13. Rao SSP, Huntley MH, Durand NC, Stamenova EK, Bochkov ID, Robinson JT, et al. A 3D map of the human genome at kilobase resolution reveals principles of chromatin looping. Cell. Elsevier Inc.; 2014;159: 1665–1680. doi:10.1016/j.cell.2014.11.021

14. Ajiro M, Jia R, Yang Y, Zhu J, Zheng Z-M. A genome landscape of SRSF3-regulated splicing events and gene expression in human osteosarcoma U2OS cells. Nucleic Acids Res. 2016;44: 1854–70. doi:10.1093/nar/gkv1500

15. Kent WJ. BLAT---The BLAST-Like Alignment Tool. Genome Res. Cold Spring Harbor Laboratory Press; 2002;12: 656–664. doi:10.1101/gr.229202

16. Breslauer KJ, Frank R, Blöcker H, Marky L a. Predicting DNA duplex stability from the base sequence. Proc Natl Acad Sci U S A. 1986;83: 3746–3750. doi:10.1073/pnas.83.11.3746

17. Zhou T, Yang L, Lu Y, Dror I, Dantas Machado AC, Ghane T, et al. DNAshape: a method for the high-throughput prediction of DNA structural features on a genomic scale. Nucleic Acids Res. 2013;41: W56-62. doi:10.1093/nar/gkt437
