# Supplementary material for: A machine learning approach for predicting CRISPR-Cas9 cleavage efficiencies and patterns underlying its mechanism of action
Source: PLoS Comput Biol. 2017 Oct 16;13(10):e1005807. doi: 10.1371/journal.pcbi.1005807 (PMC5658169; doi:10.1371/journal.pcbi.1005807)
Supplement: S4 Table — Number of targets in each study for which NGG or NAG PAMs were found following the pairwise alignment. (DOCX) [file pcbi.1005807.s017.docx]

**S4 Table. Summary of samples for which the PAM was corrected.** Number of targets in each study for which NGG or NAG PAMs were found following the pairwise alignment.

|  | Non-NGG original | Gained NGG PAM | Gained NAG PAM | Remained NAG^a^ | Remained other^b^ |
| --- | --- | --- | --- | --- | --- |
| Tsai data | 54 | 20 | 13 | 12 | 9 |
| Kleinstiver data | 31 | 8 | 9 | 11 | 3 |
| Frock data | 34 | 17 | 5 | 8 | 4 |

^a^The number of targets with an NAG PAM, that were not corrected to an NGG PAM.
^b^The number of targets with a non-canonical PAM (NGG or NAG) that remained so following the pairwise alignment procedure.
