# Supplementary material for: A machine learning approach for predicting CRISPR-Cas9 cleavage efficiencies and patterns underlying its mechanism of action
Source: PLoS Comput Biol. 2017 Oct 16;13(10):e1005807. doi: 10.1371/journal.pcbi.1005807 (PMC5658169; doi:10.1371/journal.pcbi.1005807)
Supplement: S6 Table — The averaged Pearson r2 was re-computed while altering the sample size of this set from 100% to 0% (relative to the size of the set of the cleaved sites). (DOCX) [file pcbi.1005807.s019.docx]

**S6 Table. Averaged Pearson *r^2^* values for reduced sizes of the set of uncleaved sites.** The averaged Pearson *r^2^* was re-computed while altering the sample size of this set from 100% to 0% (relative to the size of the set of the cleaved sites).

| Size of the set of sampled uncleaved sites  (proportional to the size of the set of cleaved sites) | Pearson *r*^2^ | | | |
| --- | --- | --- | --- | --- |
|  | CRISTA | CCTop | OptCD | CFD score |
| 0% | 0.48 | 0.23 | 0.29 | 0.42 |
| 25% | 0.74 | 0.44 | 0.31 | 0.56 |
| 50% | 0.77 | 0.48 | 0.31 | 0.6 |
| 75% | 0.8 | 0.48 | 0.32 | 0.64 |
| 100% (as presented in the main text) | 0.8 | 0.46 | 0.32 | 0.65 |
