# Supplementary material for: A machine learning approach for predicting CRISPR-Cas9 cleavage efficiencies and patterns underlying its mechanism of action
Source: PLoS Comput Biol. 2017 Oct 16;13(10):e1005807. doi: 10.1371/journal.pcbi.1005807 (PMC5658169; doi:10.1371/journal.pcbi.1005807)
Supplement: S8 Table — The order of features indicates the selection order. The accuracy measurements (RMSE, Pearson r2, ROC AUC, and PRC AUC) in each row are computed over the model when trained on the incremental set of features that were selected until that point. (DOCX) [file pcbi.1005807.s021.docx]

**S8 Table. The top 30 selected features in the forward selection procedure.** The order of features indicates the selection order. The accuracy measurements (RMSE, Pearson r2, ROC AUC, and PRC AUC) in each row are computed over the model when trained on the incremental set of features that were selected until that point.

| **Feature (order of selection)** | **RMSE** | **Pearson *r^2^*** | **ROC AUC** | **PRC AUC** |
| --- | --- | --- | --- | --- |
| pairwise alignment score | 1.322101 | 0.772779 | 0.959808 | 0.946456 |
| PAM type | 1.218246 | 0.801116 | 0.972539 | 0.957077 |
| #wobble total | 1.189188 | 0.809254 | 0.978297 | 0.965419 |
| #RNA bulges | 1.166092 | 0.817769 | 0.980908 | 0.966847 |
| #mismatches in positions 17-20 | 1.155 | 0.818346 | 0.98 | 0.968338 |
| #mismatches | 1.162968 | 0.817539 | 0.980251 | 0.968747 |
| #DNA bulges | 1.164569 | 0.817341 | 0.980367 | 0.968831 |
| 20th position nucleotide | 1.161693 | 0.817413 | 0.983906 | 0.970873 |
| DNA enthalpy – extended 223nt | 1.15538 | 0.823567 | 0.984762 | 0.972156 |
| MGW (minor groove width) at the PAM NNGGN | 1.090462 | 0.836159 | 0.985501 | 0.97389 |
| nucleotide - position 2 | 1.090863 | 0.840182 | 0.985527 | 0.974364 |
| #Tv (transversion mismatches) total | 1.088334 | 0.842017 | 0.986118 | 0.975386 |
| DHS (DNAse hypersensitive site) signal value | 1.085552 | 0.843223 | 0.986408 | 0.975437 |
| #RR (purine-purine) total | 1.094655 | 0.843302 | 0.985955 | 0.975088 |
| in exon (non-NGG strand) | 1.098063 | 0.842331 | 0.986079 | 0.975222 |
| Guanine occupancy | 1.107687 | 0.842014 | 0.985627 | 0.974822 |
| #YY (pyrimidine-pyrimidine) total | 1.103198 | 0.841498 | 0.985694 | 0.97445 |
| downstream nt - position 1 | 1.106476 | 0.841521 | 0.986454 | 0.975916 |
| downstream nt - position 5 | 1.10993 | 0.841768 | 0.986447 | 0.975901 |
| distance from nucleosome | 1.123196 | 0.841495 | 0.986507 | 0.976156 |
| nucleotides - positions 4-5 | 1.135493 | 0.841614 | 0.986164 | 0.97556 |
| downstream nt - position 2 | 1.14111 | 0.841041 | 0.986241 | 0.975707 |
| transcription region | 1.149159 | 0.840246 | 0.986362 | 0.975859 |
| coding region | 1.159097 | 0.839248 | 0.98635 | 0.97576 |
| GC content - extended | 1.14066 | 0.840285 | 0.985968 | 0.975411 |
| in exon (NGG strand) | 1.149718 | 0.839493 | 0.986051 | 0.975559 |
| nucleotide - position 4 | 1.153458 | 0.838713 | 0.985888 | 0.975125 |
| NGG strand expression | 1.159924 | 0.837902 | 0.985804 | 0.975037 |
| non-NGG strand expression | 1.165366 | 0.837195 | 0.986007 | 0.975185 |
| PAM N nucleotide | 1.1718 | 0.836294 | 0.98596 | 0.975116 |
